# Leptin Induces Proadipogenic and Proinflammatory Signaling in Adipocytes

**DOI:** 10.3389/fendo.2019.00841

**Published:** 2019-12-13

**Authors:** Lohanna Palhinha, Sally Liechocki, Eugenio D. Hottz, Jéssica Aparecida da Silva Pereira, Cecília J. de Almeida, Pedro Manoel M. Moraes-Vieira, Patrícia T. Bozza, Clarissa Menezes Maya-Monteiro

**Affiliations:** ^1^Laboratory of Immunopharmacology, Oswaldo Cruz Institute (IOC), Oswaldo Cruz Foundation (FIOCRUZ), Rio de Janeiro, Brazil; ^2^Laboratory of Glycoconjugates Analysis, Department of Biochemistry, Federal University of Juiz de Fora (UFJF), Juiz de Fora, Brazil; ^3^Laboratory of Immunometabolism, Department of Genetics, Evolution, Microbiology and Immunology, Institute of Biology, University of Campinas, Campinas, Brazil; ^4^Post-Graduate Program in Immunology, Institute of Biological Sciences, University of Sao Paulo, São Paulo, Brazil; ^5^Experimental Medicine Research Cluster, EMRC, University of Cammpinas, Campinas, Brazil

**Keywords:** adipogenesis, leptin, insulin, lipid droplet, adipose tissue, adipose-derived stromal cells, preadipocyte differentiation, mTOR

## Abstract

**Background:** Leptin is an adipokine with well-known effects on the central nervous system including the induction of energy expenditure and satiety. Leptin also has major relevance when activating immune cells and modulating inflammatory response. In obesity, increases in white adipose tissue accumulation and leptin levels are accompanied by hypothalamic resistance to leptin. Even though the adipose tissue is a leptin-rich environment, the local actions of leptin regarding adipogenesis were not thoroughly investigated until now. Here we evaluate the contributions of leptins direct signaling in preadipocytes and adipose tissue-derived stromal cells (ASCs) for adipogenesis.

**Methods:** Adipocytes were differentiated from the murine lineage of preadipocytes 3T3-L1 or ASCs from subcutaneous and visceral (retroperitoneal) fat depots from C57Bl/6J mice. Differentiating cells were treated with leptin in addition to or in replacement of insulin. The advance of adipogenesis was assessed by the expression and secretion of adipogenesis- and lipogenesis-related proteins by Western blot and immunoenzimatic assays, and the accumulation of lipid droplets by fluorescence microscopy.

**Results:** Leptin treatment in 3T3-L1 preadipocytes or ASCs increased the production of the adipogenesis- and lipogenesis-related proteins PLIN1, CAV-1, PPARγ, SREBP1C, and/or adiponectin at earlier stages of differentiation. In 3T3-L1 preadipocytes, we found that leptin induced lipid droplets' formation in an mTOR-dependent manner. Also, leptin induced a proinflammatory cytokine profile in 3T3-L1 and ASCs, modulating the production of TNF-α, IL-10, and IL-6. Since insulin is considered an essential factor for preadipocyte differentiation, we asked whether leptin would support adipogenesis in the absence of insulin. Importantly, leptin induced the formation of lipid droplets and the expression of adipogenesis-related proteins independently of insulin during the differentiation of 3T3-L1 cells and ASCs.

**Conclusions:** Our results demonstrate that leptin induces intracellular signaling in preadipocytes and adipocytes promoting adipogenesis and modulating the secretion of inflammatory mediators. Also, leptin restores adipogenesis in the absence of insulin. These findings contribute to the understanding of the local signaling of leptin in precursor and mature adipose cells. The proadipogenic role of leptin unraveled here may be of especial relevance during obesity, when its central signaling is defective.

**Graphical Abstract:**
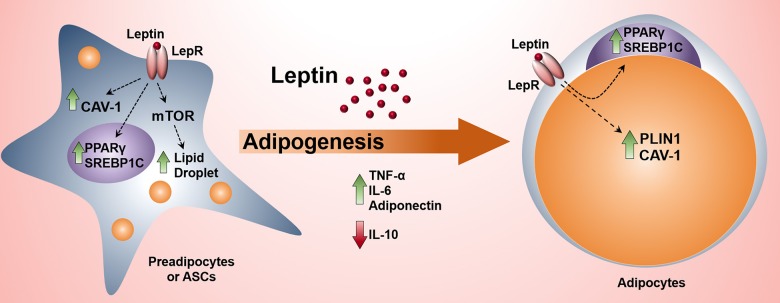
The activation of leptin signaling pathway in preadipocytes induces adipogenesis and the production of proinflammatory cytokines.

## Highlights

- Leptin anticipates adipocyte differentiation in an mTOR-dependent manner.
- Leptin sustains adipogenesis of 3T3-L1 and ASCs in the absence of insulin.
- Leptin induces a proinflammatory cytokine profile in adipocytes.

## Introduction

According to the World Health Organization, obesity prevalence nearly tripled since 1975 (1). Obesity is characterized by the expansion of the white adipose tissue with a subsequent imbalance in the production and signaling of adipokines accompanied by the development of chronic low-grade inflammation (2). Due to the obesity-associated inflammation and metabolic reprogramming, obese individuals have increased risk of developing comorbidities such as cancer, type II diabetes, stroke, among others (1, 3, 4). Thus, the understanding of the onset of obesity is of major importance, including adipogenesis itself.

The white adipose tissue (WAT) is recognized as an essential immunoendocrine organ that controls energy balance and metabolism (5). Adipocytes, the main cell type within adipose tissue, secrete hormones/cytokines collectively classified as adipokines. The first-described adipokine was leptin (6), one of the first links between the adipose tissue and the neuroendocrine control of energy homeostasis (6, 7). Leptin binds to the long leptin receptor isoform (LepRb), leading to the activation of JAK2/STAT3 and PI3K/AKT/mTOR pathways (8–10). In the hypothalamus, activation of the mTOR pathway by leptin is important for the induction of energy expenditure and satiety and consequently, a long-term effect on preventing excessive WAT accumulation (10). On the other hand, mTOR has been shown to be essential for adipogenesis (11, 12). We have previously shown that leptin can induce formation of lipid droplets in an mTOR-dependent manner, in different cell types (13–15). In peripheral tissues, leptin participates in reproduction (16), activation of immune cells (15, 17, 18), cell proliferation (14), osteogenesis (19), among many other functions. Leptin levels are increased during obesity but leptin signaling in the hypothalamus is impaired, a phenomenon called central leptin resistance (9, 20, 21). The concentration of leptin in the white adipose tissue depots may be much higher than its circulating serum levels. Although the hypertrophy and hyperplasia of adipocytes occur in this leptin-rich environment, leptin's paracrine, and autocrine effects on adipocyte differentiation are still not clear.

Here we tested leptin's ability to modulate adipogenesis in 3T3-L1 and primary adipose-derived stromal cells (ASCs) of subcutaneous and visceral WAT depots. Our data show that leptin is able to accelerate the differentiation of preadipocytes. Further, we describe that leptin induces adipogenesis even in the absence of insulin—which is considered an essential hormone for the induction of adipogenesis (22). The results presented here indicate an unexpected new role for leptin as a proadipogenic factor.

## Materials and Methods

### 3T3-L1 Preadipocyte Differentiation

The murine lineage of preadipocytes NIH3T3-L1 was obtained from the National Bank of Cells, Federal University of Rio de Janeiro, Brazil. To keep preadipocytes in their undifferentiated state, we cultured the cells with medium containing Dulbecco's Modified Eagle Medium (DMEM) (Gibco) with 4.5 g/L of glucose (Invitrogen) supplemented with penicillin (100 U/mL) and streptomycin (100 μg/mL) (Gibco) and with 10% of bovine serum (Invitrogen). Preadipocytes (1.05 × 10^4^ cells/cm^2^ of adherence area) were seeded in polystyrene culture plates (Biofil). Four days after the seeding (day 0) medium was replaced by the differentiation-inducting medium containing DMEM with 4.5 g/L of glucose, penicillin (100 U/mL), streptomycin (100 μg/mL), 10% of fetal bovine serum, 1 μM of dexamethasone (Sigma Aldrich), 0.5 mM of isobutylmethylxantine (IBMX) (Sigma Aldrich) and 0.3 units of insulin/mL (Regular Humulin—Lily). The differentiation induction medium was maintained for 3 days and then replaced by the maturation medium, containing DMEM with 4.5 g/L of glucose, penicillin (100 U/mL), streptomycin (100 μg/mL), 10 % of fetal bovine serum and 0.3 units/mL of insulin. Seventy-five percent of the maturation medium was renewed every 2–3 days.

For the experiments in which cells were differentiated in the presence of leptin, the first supplementation with leptin [4 or 40 nM, doses approximately matched to the circulating levels of leptin in obese and morbidly obese individuals (23–25)] occurred at day−3 (1 day after plating) and leptin was added at every medium renewal.

To investigate the ability of leptin to induce preadipocyte differentiation in the absence of insulin, insulin was replaced by leptin at day 0 and differentiation was performed as aforementioned.

### Murine Mesenchymal Stromal Cells (ASCs) Isolation and Differentiation

Subcutaneous and retroperitoneal fat pads were isolated from male C57Bl/6J mice of 6 weeks of age. In order to achieve a considerable amount of stem cells to expand and differentiate we pooled right and left tissue depots from three animals for each experimental replicate. For the experiments using LepR-deficient mouse, we pooled the right and left tissue depots (subcutaneous, retroperitoneal and epididymal) from one BKS.Cg-m^+^/^+^Lepr^db^/JUnib male mouse (UNICAMP, Campinas, SP, Brazil). Tissues were washed with phosphate-buffered saline (PBS) solution with penicillin (100 U/mL) and streptomycin (100 μg/mL) (Sigma) and 5 μg/mL of ciprofloxacin (Sigma) and then cut into small pieces. These pieces were rewashed with the same solution and incubated for 2 h with 2 mg/mL of collagenase (Sigma) in a volume of 2.5 mL per mg of tissue with constant shaking at 37°C. Collagenase was inactivated by doubling the volume with media containing antibiotics and 10% fetal bovine serum (Life Sciences). Digested tissues were then filtered in cell strainers of 100 μm pore size and centrifuged at 500 × g for 7 min. The superior fraction which contained mainly lipids and mature adipocytes was discarded and the pellet was resuspended in PBS plus penicillin (100 U/mL) and streptomycin (100 μg/mL), 5 μg/mL of ciprofloxacin, filtered again in cell strainers of 40 μm pore size and centrifuged at 500 × *g* for 7 min. The pellet of stromal vascular cells was then resuspended in culture media containing DMEM with 4.5 g/L glucose, penicillin (100 U/mL) and streptomycin (100 μg/mL), 5 μg/mL of ciprofloxacin, and 20% of fetal bovine serum (Life Sciences) and cultured. Cells were expanded 3–4 times before plating. All animal procedures were approved by the Committee of Ethics in Animal Research L011.2015.

### Characterization of ASCs by Flow Cytometry

Stromal vascular cells expanded up to two times were labeled with ASCs' positive (CD44, CD29, CD106, and CD105) and negative (MHC-class II, CD11b, CD31, CD45, and CD144) markers. Cells were incubated (30 min) with FITC-conjugated anti-CD45 (eBioscience, cat 12-1051-81, dilution 1:20); -CD31 (eBioscience, cat 11-0311-81, dilution 1:20) and -MHC class II (eBioscience, cat: 11-5320-82, dilution 1:20); APC-conjugated anti-CD11b (BD Pharmingen, cat 553312, dilution 1:20), and PE-conjugated anti-CD29 (eBioscience, cat 12-0291-81, dilution 1:20) or -CD105 (eBioscience, cat 12-1051-81, dilution 1:10). For evaluation of CD106 expression, cells were incubated (30 min) with rat anti-mouse CD106 (eBioscience, cat 14-1061-81, dilution 1:10) followed by 30 min incubation with Alexa Fluor® 546-conjugated anti-rat IgG (Molecular Probes, cat: A-11081, dilution 1:250); unbound antibodies were washed out and cells were incubated (30 min) with FITC-conjugated anti-CD45, -CD31, and -MHC class II, and APC-conjugated anti-CD11b. For evaluation of CD44 (eBioscience, cat 11-0441-81, dilution 1:20) expression, cells were incubated (30 min) with unconjugated rat antibodies against CD45 (BD Biosciences, cat: 550539, dilution 1:10) and CD144 (eBioscience, cat: 16-1441-85, dilution 1:20) followed by 30 min incubation with AlexaFluor 546-conjugated anti-rat IgG; unbound antibodies were washed out and cells were incubated (30 min) with FITC-conjugated anti-CD44 and APC-conjugated anti-CD11b antibodies. Cells incubated with isotype-matched IgG conjugated with the same fluorochromes or unconjugated IgG followed by incubation with the secondary antibody were used as a negative control. Cells were acquired in a Beckman Coulter CytoFLEX S using CytExpert software and analyzed using FlowJo v10 software. For analysis, cells were gated by the exclusion of leucocytes and endothelial cells markers (CD45, MHC class II, CD11b, CD31, and CD144) and the expression of ASCs markers evaluated as shown in Supplementary Figure 3.

### Fluorescence Microscopy Analysis

Cells were fixed for 15 min with formaldehyde 3.7 %, washed with buffered saline, and stained with BODIPY^TM^ 493/503 (ThemoFisher Scientific) for 30 min and DAPI (ThemoFisher Scientific) for 5 min. Images were acquired with the microscope Olympus BX60 and analyzed with the software Fiji (26) version 1.49 m (National Institutes of Health, USA) with Java version 1.6.0_24 (64-bit). We developed a macro to analyze the total Bodipy stained area (green) in each field adjusting the same parameters of color balance, contrast, background, and noise. Images were processed so that the threshold setting for quantifying the total and relative area of Bodipy staining excluded most of the interferences from the image acquisition. A different macro was developed for the counting of nuclei numbers in each field (DAPI—blue). Then, total Bodipy stained area was normalized by the number of cells in each field and the mean of these measurements was plotted for each group.

### Western Blot Analysis

Cells were washed with phosphate-buffered saline (PBS) solution and then subjected to lysis directly by 95°C-heated Laemmli buffer with concomitant cell scraping from the wells. Protein content was separated by 10–15% sodium dodecyl sulfate-polyacrylamide gel electrophoresis (SDS-PAGE) and transferred to nitrocellulose membranes. Membranes were blocked with 5% non-fat dried milk diluted in Tris-Buffered Saline supplemented with 0.1% Tween 20 (Sigma) (TBS-T) for 1 h before incubation overnight with primary antibodies against PLIN1 (Cell Signaling, cat 3470, dilution 1:500), JAK2 (Cell Signaling, cat 3230, dilution 1:1,000), pTyr1007/1008 JAK2 (Cell Signaling, cat 3771, dilution 1:1,000), pTyr389 S6K (Cell Signaling, cat 9205, dilution 1:1,000), S6K (Cell Signaling, cat 2708, dilution 1:1,000), pTyr705 STAT3 (Cell Signaling, cat 9131, dilution 1:1,000), STAT3 (Cell Signaling, cat 9139, dilution 1:1,000), PPARγ (Santa Cruz, cat sc-7196, dilution 1:1,000), SREBP1 (Santa Cruz, cat sc-13551, dilution 1:1,000), and for 1–2 h with anti-β-actin (Sigma, cat A2228, dilution 1:10,000) or CAV-1 (Santa Cruz, cat sc-894, dilution 1:2,000). Membranes were then washed with TBS-T and proteins were detected using fluorescent dye-conjugated secondary antibodies (anti-mouse, IRDye 800CW, cat 926-32210 or anti-rabbit, IRDye 680RD cat 926-68071, LI-COR) or horseradish peroxidase-conjugated secondary antibodies (anti-rabbit, cat PI-1000 or anti-mouse, cat PI-2000; Vector).

### Quantification of Cytokines

Supernatants were harvested from cultured preadipocytes and adipocytes or differentiating ASCs at different timepoints and the levels of leptin, adiponectin, TNF-α, IL-10, IL-6, KC/CXCL1, and MCP-1were measured using standard ELISA protocols according to manufacturer's instructions (R&D Systems).

### Statistical Analysis

Statistics were performed using GraphPad Prism (San Diego, CA) version 6.05. All numeric variables were tested for normal distribution using the Kolmogorov-Smirnov normality test. For comparison among three or more groups, we used One-way ANOVA with Dunnet's post-test to compare differentiated cells with preadipocytes, or Tukey's post-test to compare leptin-treated with untreated cells among groups following a normal distribution. In cases of non-parametric distribution among three or more groups, we used One-way ANOVA, followed by Kruskal-Wallis' test with Dunn's correction. To analyze the effect of rapamycin over the effect of leptin we used Two-way ANOVA with Sidak's post-test to locate the differences among groups. For comparison between two groups we used the Mann-Whitney *U*-test for non-parametric distribution. For comparisons when the groups were normalized by the control group (thus containing repeated value) we used the Kolmogorov-Smirnov non-parametric test.

## Results

### Leptin Increases Preadipocyte Differentiation

**Figure 1:**
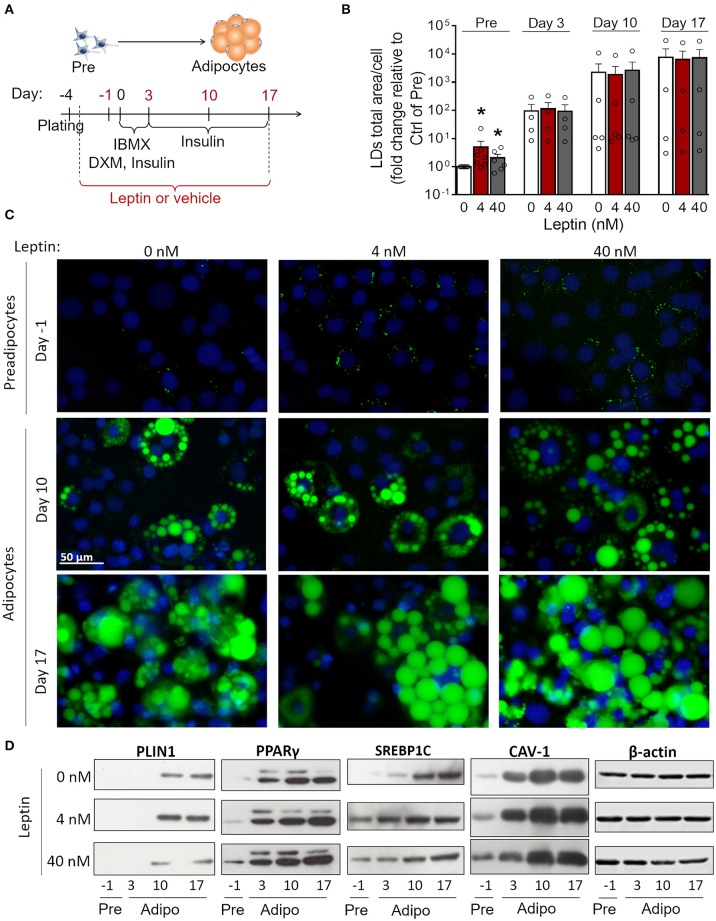
Leptin treatment enhances the expression of adipogenesis- and lipogenesis-related proteins. **(A)** Schematic representation of experimental design: leptin was administered from day−3 up to days−1, 3, 10, or 17 of differentiation. Day 0 represents the day of the induction of differentiation (with IBMX – isobutylmethylxantine, DXM—Dexamethazone and insulin). **(B,C)** Preadipocytes (day−1) and adipocytes (days 3–17) were differentiated with leptin (4 or 40 nM) and stained with Bodipy (green) for lipid droplets and DAPI (blue) for nuclei. **(B)** Quantification of the green area per cell in each condition. Bars represent mean ± standard error of the mean of 4–7 independent experiments. *Means *p* < 0.05 in a Kolmogorov-Smirnov non-parametric test. **(C)** Representative images of 4–7 independent experiments. Scale bar represents 50 μm in range. All images have the same dimensions. **(D)** Western blot analysis of PLIN1, PPARγ, SREBP1C, caveolin-1 (CAV-1), and β-actin in 3T3-L1 preadipocytes and adipocytes differentiated with or without leptin. Blots are representative of 3–5 independent experiments. Lysates from cells differentiated with 0, 4, or 40 nM were analyzed in the same gel and cropped for clearer comparison among groups.

**Figure 2:**
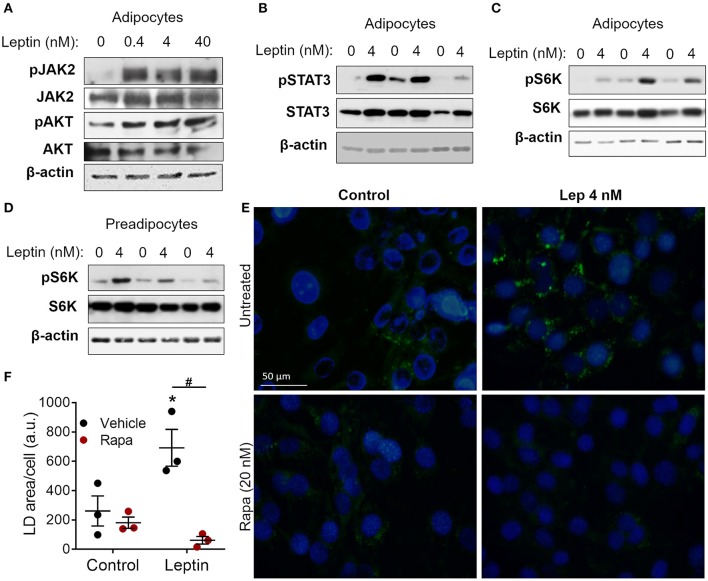
Leptin-induced adipogenesis depends on mTOR/p70 S6K pathway. **(A–D)** 3T3-L1 cells were cultured as described in methods up to day−1 (preadipocytes) and day 17 (adipocytes) and then stimulated with leptin at the indicated concentrations for 20 min. **(A)** Western blot analysis of phosphorylated JAK2 (pJAK2), total JAK2, pAKT, total AKT, and β-actin. Blots are representative of three independent experiments. **(B)** Western blot analysis of pSTAT3, total STAT3, and β-actin in adipocytes from three independent experiments analyzed in the same gel. **(C,D)** Western blot analysis of pS6K, total S6K, and β-actin in adipocytes **(C)** and preadipocytes **(D)** from three independent experiments. **(E,F)** Preadipocytes were cultured for 48 h with no stimulus (control), leptin (4 nM), rapamycin (20 nM), or both. Cells were stained with Bodipy (green) for lipid droplets and with DAPI (blue) for nuclei. **(E)** Shows images representative of three independent experiments. Scale bar represents 50 μm in range. **(F)** Quantification of the green area per cell in each condition. Dots represent the replicates of one experiment representative of three. Horizontal lines represent mean ± standard error of the mean. *Means *p* < 0.05 relative to control, ^#^means *p* < 0.05 between vehicle and rapamycin treated cells. Statistical analyzes were performed by using Two-way ANOVA with Sidak's post-test.

3T3-L1 cells progressively differentiated into adipocytes up to 17 days after the induction of adipogenesis (day 0) with increased formation of lipid droplets accompanied by enhanced secretion leptin and adiponectin (Supplementary Figures 1A–D). Briefly, the adipocyte differentiation protocol (Figure 1A) consisted of plating cells at day−4, followed by administration of the adipogenic medium (insulin, IBMX, dexamethasone) at day 0—so called for being the start-point of adipogenesis *in vitro*—which was maintained until day 3. Afterwards, cells were incubated in insulin-containing medium and kept in this medium until the end of the assay (Figure 1A) as described in more details in section Materials and Methods. In order to evaluate the effects of leptin in the adipogenic process, 3T3-L1 cells were incubated with leptin supplementation from the first day post-plating (day−3) until the end of the differentiation protocol (day 17). The cells were analyzed at the preadipocyte stage (day−1) and at the three stages of adipocyte maturation (days 3, 10, or 17) (Figure 1A). Leptin significantly enhanced lipid accumulation in preadipocytes, showing an anticipation of this adipogenic feature when compared to the control cells, without leptin (Figures 1B,C). Leptin lead to increased expression of the lipid droplet protein PLIN1 in adipocytes (Figure 1D). In addition, preadipocytes and adipocytes cultured with leptin exhibited increased levels of the adipogenesis- and lipogenesis-related factors PPARγ, SREBP1C and Caveolin-1 (CAV-1), mainly in preadipocytes and adipocytes at the first stages of differentiation (Figure 2D; Supplementary Figure 2 shows the replicates of all Western blots). These results show a synergistic proadipogenic effect of leptin and insulin.

### Leptin-Induced Adipogenesis Depends on the mTOR Pathway

Leptin binds to its LepRb receptor and activates JAK2, which can activate STAT3 and PI3K/AKT/mTOR pathways (9, 10, 15). To dissect leptin signaling pathways in adipocytes, differentiated adipocytes were stimulated with leptin for 20 min and the phosphorylated proteins of each pathway were analyzed through Western blot. We observed increased phosphorylation of JAK2, AKT, and STAT3 in adipocytes after stimulation with leptin (Figures 2A,B; Supplementary Figure 2), confirming the activation of the two branches of the leptin signaling pathway in adipocytes. Leptin also induced the phosphorylation of p70 S6 kinase (S6K), a substrate of mTOR (Figure 2C). Considering the importance of mTOR signaling in the induction of adipogenesis (11, 12, 27, 28), we asked whether leptin can modulate this pathway in undifferentiated cells (Figure 2D). We found that leptin also activated the mTOR pathway in preadipocytes, as evidenced by the increased phosphorylation of p70 S6K (Figure 2D). To gain insights into the role of mTOR signaling in leptin-induced adipogenesis, we stimulated preadipocytes with leptin in the presence of rapamycin, a selective mTOR inhibitor, and evaluated the biogenesis of lipid droplets after 48 h. Rapamycin treatment completely abolished the leptin-induced biogenesis of lipid droplets in preadipocytes (Figures 2E,F), indicating that the pro-adipogenic effects of leptin depend on mTOR activation.

### Leptin Shifts Adipocyte Cytokine Production Toward a Proinflammatory Pattern

**Figure 3:**
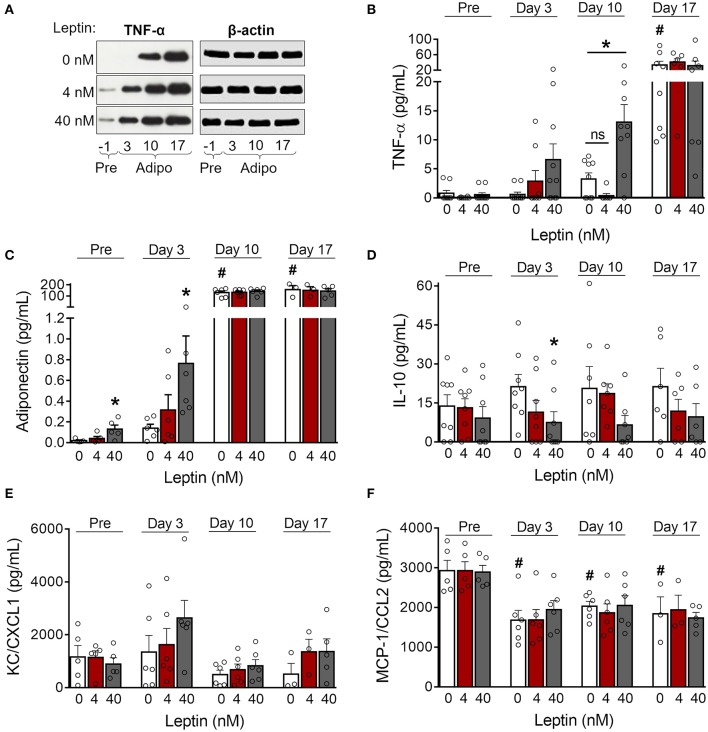
Leptin induces a proinflammatory cytokine profile during adipocyte differentiation. **(A,B)** 3T3-L1 preadipocytes (day−1) and adipocytes at days 3, 10, and 17 were cultured in the presence of leptin (0, 4, or 40 nM). **(A)** Western blot analysis of TNF-α and β-actin in cell lysates from each condition. Blots are representative of three independent experiments. Cell lysates from a representative experiment were analyzed in the same gel and cropped for clearer comparison among the groups. **(B–F)** Concentration of **(B)** TNF-α, **(C)** adiponectin, **(D)** IL-10, **(E)** KC/CXCL1, and **(F)** MCP-1/CCL2 in the supernatant of preadipocytes and adipocytes differentiated under distinct leptin stimulus. Bars represent mean ± standard error of the mean of 3–11 independent experiments. ^#^*p* < 0.05 compared to preadipocytes (day−1). *Represents *p* < 0.05 between leptin-treated and untreated cells at the same stage of differentiation. Statistical analyzes were performed by using **(B–E)** One-way ANOVA followed by Kruskal-Wallis' test with Dunn's correction or **(F)** One-way ANOVA with Dunnet's post-test. “ns” stands for non-significant.

Adipose tissue is an important source of cytokines and chemokines, which are collectively called adipokines (29). We observed increased production of TNF-α in 3T3-L1 control cells as adipogenesis progressed (Figures 3A,B; Supplementary Figure 2). Treatment with leptin further increased TNF-α expression by preadipocytes (day−1) and immature adipocytes (day 3) (Figure 3A; Supplementary Figure 2). In addition, treatment with leptin (40 nM) also induced the secretion of TNF-α and adiponectin at earlier stages of differentiation (Figures 3B,C), which is consistent with the anticipation of adipogenesis described above. Also, leptin treatment decreased the secretion of the anti-inflammatory cytokine IL-10 (Figure 3D). The levels of the chemokines KC/CXCL1 and MCP-1/CCL2, on the other hand, were not modulated by leptin treatment (Figures 3E,F). These results suggest that, in addition to adipogenesis, leptin induces a proinflammatory cytokine balance in 3T3-L1 adipocytes.

### Adipocyte Differentiation in the Absence of Insulin Is Recovered by Leptin

**Figure 4:**
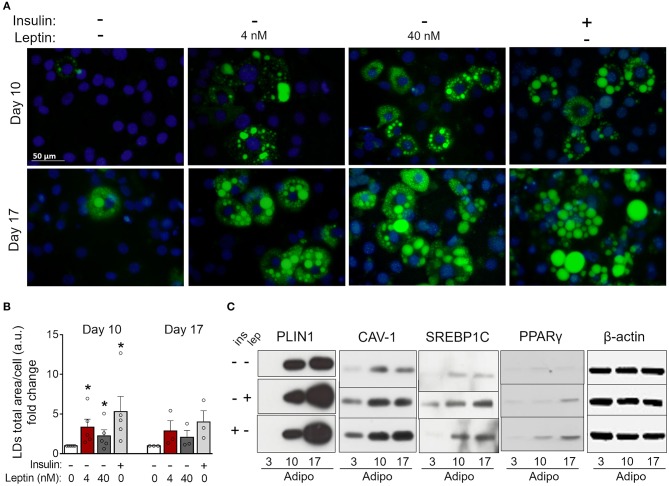
Leptin recovers 3T3-L1 differentiation in the absence of insulin. 3T3-L1 adipocytes were cultured with no hormone, insulin or leptin (4 or 40 nM) up to 3, 10, or 17 days after induction of differentiation. **(A)** Fluorescence images of cells stained with Bodipy (green) for lipid droplets and DAPI (blue) for nuclei. Images are representative of 3–5 independent experiments. Scale bar represents 50 μm. **(B)** Quantification of the green area per cell in each field. Data are depicted as the fold change relative to the group with no hormone supplementation. Bars represent mean ± standard error of the mean of 3–5 independent experiments. **(C)** Western blot analysis of PLIN1, CAV-1, SREBP1C, PPARγ, and β-actin. Blots are representative of three independent experiments in the cases of PLIN1, CAV-1, and PPARγ and two independent experiments in the case of SREBP1C. Cell lysates were analyzed in the same gel and cropped for clearer comparison among groups. *p < 0.05 relative to the group in which cells were cultured with neither insulin nor leptin. Statistical analyzes were performed by using the Kolmogorov-Smirnov test.

Insulin is considered essential for the proper differentiation of preadipocytes due to the induction of cell growth and fatty acid synthesis and is an important constituent of adipogenic cocktails (22, 30). Considering the proadipogenic effects of leptin observed above, we investigated whether leptin, in the absence of insulin, would be sufficient for the induction of adipogenesis in 3T3-L1 cells. As shown in Figure 4, 3T3-L1 cells cultured without insulin or leptin supplementation showed impaired lipid droplets' formation (Figures 4A,B) and reduced expression of the adipogenesis-related proteins PLIN1, SREBP1C, PPARγ, and CAV-1 (Figure 4C; Supplementary Figure 2) compared to insulin-differentiated cells. Importantly, replacement of insulin by leptin during 3T3-L1 differentiation recovered the biogenesis of lipid droplets and the expression of adipogenesis-related proteins (Figures 4A–C; Supplementary Figure 2). These data indicate that leptin exerts proadipogenic effects independently of insulin.

### Leptin Induces Proadipogenic and Proinflammatory Signaling in Primary Adipose Tissue-Derived ASCs

**Figure 5:**
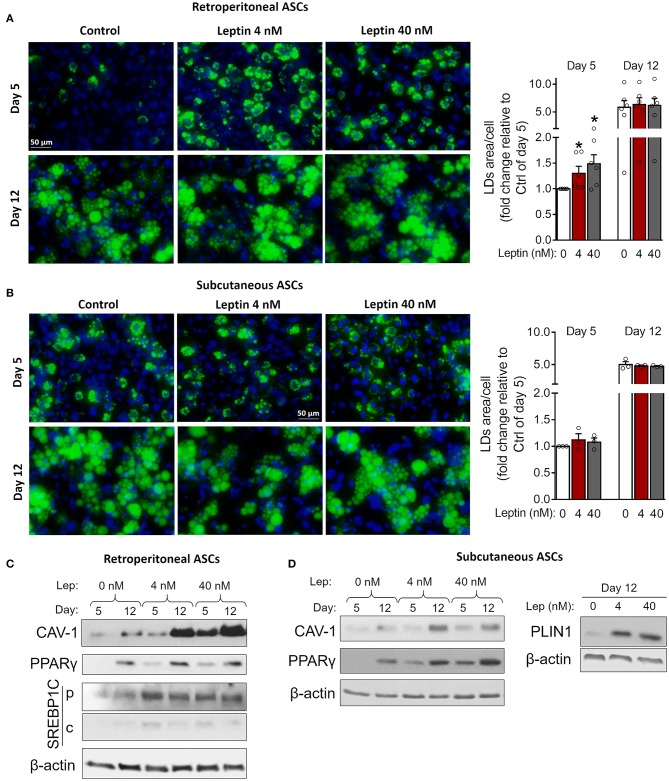
Leptin potentiates insulin-induced adipogenesis in ASCs. Adipose-derived stem cells from subcutaneous and retroperitoneal depots were isolated and then differentiated with insulin alone or insulin plus leptin (4 or 40 nM). **(A,B)** Fluorescence images of ASCs from **(A)** retroperitoneal and **(B)** subcutaneous depots that were differentiated into adipocytes and stained with Bodipy (green) for lipid droplets and with DAPI (blue) for nuclei. Images are representative of 3–6 independent experiments. Scale bar represents 50 μm. Graphs show the quantification of the green area per cell relative to the control group (insulin only) at day 5. Bars represent mean ± standard error of the mean of 3–5 independent experiments. *p < 0.05 relative to the control group of the same day. Statistical analyzes were performed by using Kolmogorov-Smirnov test. **(C,D)** Western blot analysis of CAV-1 and PPARγ and β-actin in ASCs differentiated at the conditions specified. Blots are representative of 3–5 independent experiments.

**Figure 6:**
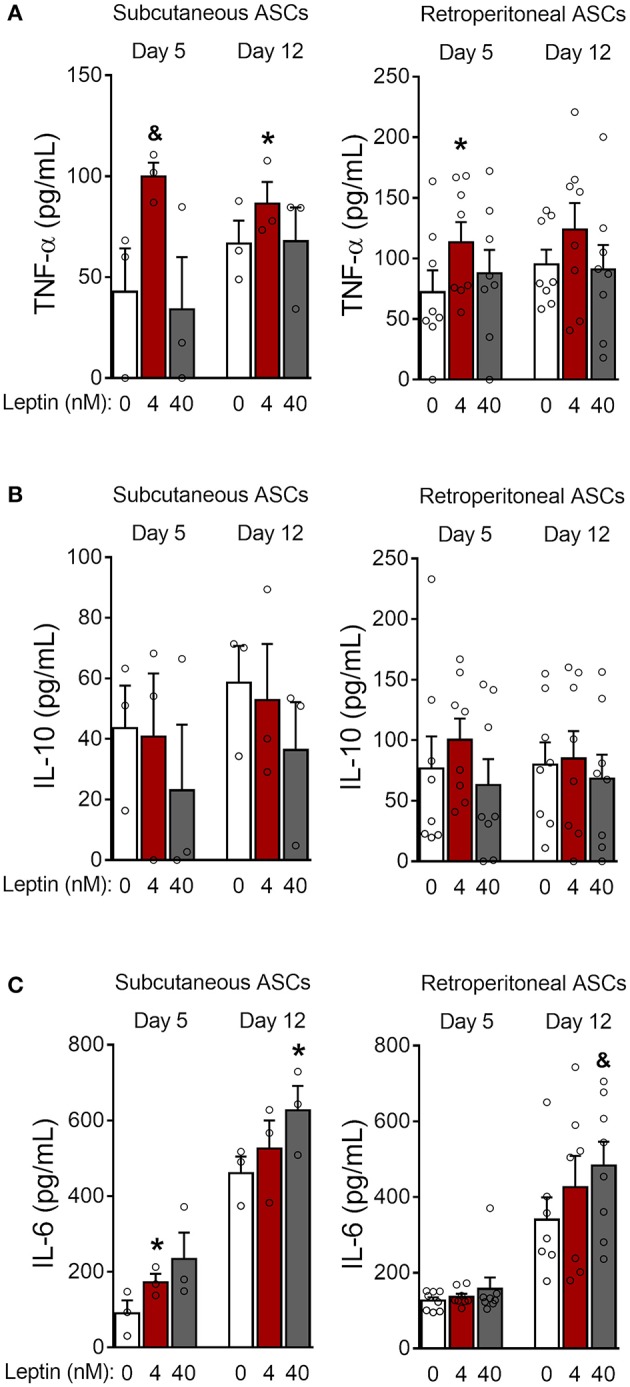
Leptin induces a proinflammatory cytokine profile during ASCs differentiation. **(A–C)** ASCs from subcutaneous and retroperitoneal fat depots were differentiated for 5 and 12 days in the presence of leptin (0, 4, or 40 nM) and the concentrations of **(A)** TNF-α, **(B)** IL-10, and **(C)** IL-6 were measured in the supernatants. Bars represent mean ± standard error of the mean of 3–8 independent experiments. *p < 0.05 and ^&^*p* < 0.06 compared to ASCs differentiated in the absence of leptin at the respective timepoint. Statistical analyzes were performed using paired *t*-test.

Next, we investigated whether leptin has proadipogenic effects in ASCs obtained from the stromal vascular fraction of subcutaneous and retroperitoneal fat pads from C57Bl/6J mice. Characterization of ASCs is described in Methods and shown in Supplementary Figure 3. Similar to 3T3-L1 cells, leptin also synergized with insulin and anticipated the differentiation of ASCs into adipocytes (Figure 5). As shown in Figures 5A,B, concomitant treatment of retroperitoneal—but not subcutaneous—ASCs with leptin and insulin increased lipid accumulation at day 5 of differentiation compared to control cells cultured with insulin only. Morphologically, ASCs cultured with insulin had already achieved maximal lipid accumulation at day 12, so there was no observable effect of leptin in potentiating insulin-induced lipid droplet biogenesis at this time point (Figures 5A,B). On the other hand, induction of adipogenesis and anticipation of differentiation were evidenced by the increased expression of CAV-1, PPARγ, SREBP1C and/or PLIN1 at days 5 and 12 in ASCs obtained from both depots (Figures 5C,D; Supplementary Figure 2). As shown with 3T3-L1, leptin treatment was able to modulate the expression of the adipogenic markers in ASCs. These data show that leptin is able to potentiate insulin-induced ASCs differentiation into adipocytes. In addition, leptin treatment induced a proinflammatory cytokine profile in differentiated ASCs by increasing the levels of TNF-α and IL-6 without affecting IL-10 levels (Figure 6).

**Figure 7:**
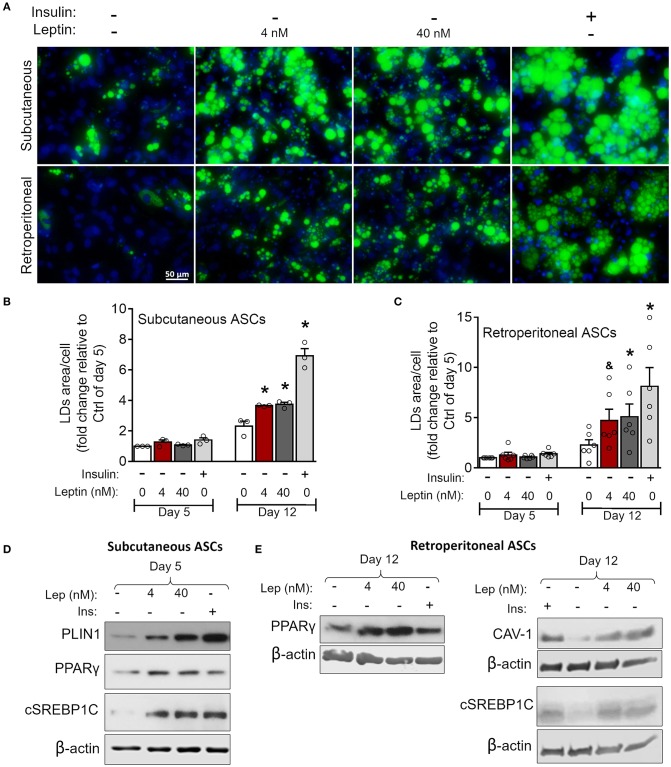
Leptin induces adipogenesis in ASCs independently on insulin. ASCs from subcutaneous and retroperitoneal depots were isolated and then differentiated up to 5 or 12 days with neither insulin nor leptin, leptin (4 or 40 nM), or insulin. **(A)** Fluorescence images of ASCs differentiated for 12 days and stained with Bodipy (green) for lipid droplets and with DAPI (blue) for nuclei. Images are representative of 3–6 independent experiments from different animal pools. Scale bar represents 50 μm. **(B,C)** Quantification of the green area per cell in adipocytes differentiated from **(B)** subcutaneous or **(C)** retroperitoneal ASCs. Graphs depict the fold change relative to the group with no hormone supplementation at day 5. Bars represent mean ± standard error of the mean of 3–5 independent experiments. *Means *p* < 0.05 and & means *p* = 0.06 relative to the control group at the same day. Statistical analyzes were performed by using One-way ANOVA with Dunnet's post-test. **(D)** Western blot analysis of PLIN1, PPARγ, cleaved SREBP1C (cSREBP1C), and β-actin in subcutaneous ASCs or **(E)** CAV-1, cSREBP1C, PPARγ, and β-actin in retroperitoneal ASCs after 12 days of differentiation in each condition. Blots are representative of 3–5 independent experiments.

As previously mentioned, adipocyte differentiation can be achieved by the combination of adipogenesis induction factors, with insulin being considered indispensable for this purpose (30). We then investigated the ability of leptin to support ASCs commitment to adipocytes in the absence of insulin. As shown in Figure 7, leptin induces lipid accumulation at day 12 of differentiation in subcutaneous and retroperitoneal ASCs when compared to the cells cultured without leptin or insulin supplementation (Figures 7A–C). Also, stimulation with leptin alone supported the expression of the adipogenesis-related proteins CAV-1, PLIN-1, PPAR-γ, and/or SREBP1C in ASCs from both adipose tissue depots (Figures 7D,E; Supplementary Figure 2). In addition, leptin did not induce adipogenesis in ASCs from LepRb-deficient (db/db) mouse, albeit these cells were still able to differentiate in response to insulin (Supplementary Figures 4A,B). Also, leptin signaling through mTOR pathway was absent in ASCs from db/db mice (Supplementary Figure 4C). Our data show that leptin supports the induction of adipocyte differentiation through LepRb signaling, even in the absence of insulin.

## Discussion

Leptin is a key adipokine in the control of energy balance and satiety through its signaling in the central nervous system (9, 22). Several effects of leptin regarding the systemic control of metabolism in various organs and systems are well-documented and still very explored by many groups (9, 31). However, one simple question remained elusive so far: what are the autocrine and paracrine effects of leptin on its own producer cells? Our data provide a new branch in leptin signaling regarding the local modulation of the adipogenic process. Here we show that leptin has direct effects in 3T3-L1 preadipocytes and adipocytes, as well as in ASCs, anticipating the commitment with the adipocyte lineage and adipogenesis.

Continuous leptin supplementation had similar effects on the differentiation of primary mouse ASCs and the murine lineage of preadipocytes 3T3-L1, anticipating and enhancing several adipogenic features. Expression of the adipogenesis- and lipogenesis-related proteins PPARγ, SREBP1C, PLIN1, and CAV-1 were enhanced both in 3T3-L1 and ASCs from subcutaneous and retroperitoneal depots. We also observed that leptin anticipated the secretion of the adipokine adiponectin, which is expressed exclusively by mature adipocytes, corroborating the advanced state of differentiation upon leptin treatment (32).

Previous studies showed that leptin induced lipolysis in rat adipose tissue (33–35). In these studies, most experiments were performed with the whole explant of WAT or isolated mature adipocytes stimulated with leptin, acutely, for 1 or 2 h (33, 35). As a matter of fact, these studies do not oppose to ours since adipogenesis is not the opposite of lipolysis: increased adipocyte differentiation only means that adipogenesis is prevailing over lipolysis in the time points observed. In other studies the cells were stimulated also for a short period and/or with high concentrations of leptin, above the physiological or obesity-associated levels (34, 36). These short incubations and the elevated doses of leptin can indeed acutely activate lipolytic pathways in mature adipocytes. There is also evidence of inhibition of lipolysis when tissue explants were incubated for longer periods (i.e., 18 h) with leptin (37). Rhee et al. (36) state that leptin stimulus inhibits rosiglitazone-induced adipogenesis in primary cells from ob/ob mice, The effects of leptin on rosiglitazone-treated cells may differ from leptin stimulation alone. Another report indicates an indirect proadipogenic effect of leptin showing that leptin inhibits the anti-adipogenic effects of vitamin D (38). Finally, in previous reports (36, 39) the cells were stimulated with leptin only after the induction of differentiation. We believe that the contact of the cells with leptin when they are still undifferentiated is a better model to evaluate the pro-adipogenic effect. The control of lipolysis in the mature adipocyte can also be regulated by infiltrating macrophages that can be present in different amounts depending on the inflammatory activation (40, 41). The activation of recruited and adipose tissue-resident immune cells—including macrophages—by leptin has been shown to induce the production of the potent prolipolytic and proinflammatory cytokine TNF-α (18, 42–44). In our work, we show that chronic leptin treatment increases the expression and secretion of TNF-α by adipocytes. Although TNF-α clearly induces lipolysis through diverse mechanisms including the inhibition of PPARγ (29, 45) and reduction of PLIN1 expression (46), we show that leptin treatment is able to significantly increase the expression of PPARγ, PLIN1, and many other features related to lipogenesis and adipogenesis. It has also been shown that leptin, TNF-α and other adipokines stimulate the expression of the proadipogenic miR-378 (47) and the adipogenesis-related miR-355 (48). We do not exclude the acute effect of leptin in the induction of lipolysis directly in mature adipocytes, but we clearly show that the proadipogenic effect prevails in a long-term incubation.

As stated above, the timing of leptin's treatment is of paramount importance for the outcomes observed in differentiated cells, with an early treatment of precursor cells leading to a proadipogenic leptin signaling. Sustaining this hypothesis, the specific knockout of LepR in already mature adipocytes did not have important effects on adipogenesis (49), while the specific knockout or knockdown of LepR in precursor cells impaired their ability to commit to the adipocyte fate (19, 50). Yue et al. (19) showed that the specific deletion of the leptin receptor LepR gene in the bone marrow-derived mesenchymal stromal cells (BM-MSCs) favored osteogenesis at the expense of adipogenesis within the bone marrow (19). Also, high fat diet increased the numbers of adipocytes in the limb bone and reduced bone volume in wild type mice, but not in mice with the deletion of LepR in BM-MSCs (19). The authors clearly show that the local signaling of leptin in bone marrow stromal precursors, during obesity, is essential for the adipocyte commitment and differentiation (19). In another work, Tencerova et al. (50) showed that LepR-positive BM-MSCs of obese individuals are more likely to differentiate into adipocytes, and that the silencing of LepR in BM-MSCs from obese individuals favored osteogenesis and reduced adipogenesis, with decreased expression of several adipogenesis-associated genes, alongside the impairment of the staining for lipid droplets (50). These results indicate that leptin's local effect may contribute *in vivo* to adipogenesis. Even though they used *in vivo* or *ex vivo* models and different sets of stromal cells, their results are in accordance with our data showing that leptin has local adipogenic effects in stromal cells. In addition, our work supports that leptin may be important throughout the adipogenic process, not only at the commitment phase.

Insulin is a classic inductor of adipogenesis and therefore is present in the majority of the adipogenesis *in vitro* protocols (22, 30). Indeed, when we remove insulin from the culture media, we see a dramatic reduction in differentiation. As aforementioned, when we differentiate cells with leptin in addition to insulin, we boost differentiation. These results may be explained by the fact that both leptin and insulin signal through PI3K/AKT/mTOR pathway (31, 51). Interestingly, here we also show that leptin sustains adipocyte maturation of cells cultured in insulin-free medium. These findings highlight that leptin can compensate for insulin absence when it comes to adipogenesis, with potential implications in pathological conditions such as types 1 and 2 diabetes. In patients with type 1 diabetes (T1D), who have little or no production of insulin, there are several metabolic alterations as hyperglycemia, weight loss and enhanced lipolysis with augmented lipotoxicity (52, 53). For these patients, insulin long-term usage often leads to unwanted side effects such as ectopic fat deposition and disabling episodes of hypoglycemia. In animal models of T1D, plasma levels of leptin are diminished compared to healthy animals (52–54). In these models, leptin administration improved survival, weight gain and glycemia, which were associated with metabolic recovery toward a reduction of hepatic gluconeogenesis, adipocyte lipolysis and diminished circulation of free fatty acids (52–54). Although leptin was not able to recover β-cell function and insulin secretion in neither of these studies, these findings point out beneficial actions of leptin in the absence of insulin that can overcome several caveats f insulin therapy. The described insulin-independent actions of leptin, especially regarding the recovery of body weight and decreased lipolysis, corroborate with our findings on the insulin-independent induction of adipocyte differentiation and maturation. Enhancement of adipogenesis may help to increase the lipid storage capacity of cells, dumping the circulation of free fatty acids and the deposition of ectopic fat *in vivo*. Similarly, the leptin analog metreleptin is the main treatment to human lipodystrophy syndromes, also improving several metabolic parameters (55). Even though metreleptin does not recover the deficiency in subcutaneous adipose tissue in lipodystrophic patients, most of these patients lack the major adipogenic pathways, including those involving CAV-1 and PLIN-1, among others. Moreover, these patients are also treated with metformin, which inhibits the mTOR pathway (55). There will be little or no possibility for leptin to induce adipogenesis and recover subcutaneous WAT in these cases, since either the major adipogenic pathways are primarily absent or inhibited by the treatment for diabetes. Specific studies should be done to determine the capacity of leptin in restoring adipogenesis within the different forms of lipodystrophy.

As previously mentioned, leptin and insulin share the activation of the mTOR pathway (31). It was shown that adipocyte-specific deletion of the mTOR gene in mice dramatically reduced white and brown fat masses but did not change body weight (11). This was explained by the ectopic fat accumulation in the heart, spleen and liver, with greater circulation of free fatty acids (11). These and other studies establish mTOR as an indispensable protein for lipid uptake (56), storage and adipogenesis (11, 57–59). With that in mind, we sought to determine if the proadipogenic effects of leptin in preadipocytes were also mediated by activation of the mTOR pathway. For this purpose, we treated 3T3-L1 preadipocytes—which had no insulin supplementation at all—with leptin in the presence or absence of rapamycin. Confirming our hypothesis, leptin effect on the increase of lipid droplet's biogenesis in preadipocytes was mediated by the mTOR signaling pathway as rapamycin treatment completely abolished lipid accumulation in leptin-treated cells.

Besides its clear role in the control of energy homeostasis and adipose tissue function, leptin is also very important in immune processes. For instance, it has been shown to participate in the activation and recruitment of immune cells, such as macrophages (15, 60), neutrophils (18), and eosinophils (13). In obesity, adipose cells secrete large amounts of immunometabolic mediators that usually cause a derange in homeostasis (2, 5, 61, 62). Adipose tissue, which includes adipocytes and stromal cells, becomes chronically inflamed with enhanced secretion of proinflammatory mediators, such as TNF-α and IL-6, and a decrease in anti-inflammatory ones, such as IL-10 (5, 63). Leptin was found to participate in the induction of proinflammatory cytokines in dendritic cells (17), T cells (17), and macrophages (15, 44, 60). In this report we show that leptin is a proinflammatory factor also in adipocytes, as shown by the increase in TNF-α and the decrease in IL-10 production by 3T3-L1 cells, and increased TNF-α and IL-6 in differentiated ASCs. Although the secretion of the chemokines KC/CXCL1 and MCP-1/CCL2 remained unchanged in 3T3-L1, our results show that leptin's actions on adipocytes contribute to the inflammatory profile characteristic of obesity.

In obesity, leptin central effects (i.e., induction of energy expenditure) is impaired, therefore its local effects may prevail and contribute to white adipose tissue expansion and enhanced inflammatory milieu. In addition, the direct induction of leptin signaling pathways in preadipocytes and adipocytes is sufficient to support adipogenesis regardless of the presence of insulin. Our data open a new perspective for the study of leptin as an inductor of adipogenesis and associated inflammation.

## Data Availability Statement

All datasets generated for this study are included in the article/Supplementary Material.

## Ethics Statement

The animal study was reviewed and approved by Committee of Ethics in Animal Research from Oswaldo Cruz Institute/CEUA-IOC L011.2015.

## Author Contributions

All authors had critically revised and approved the final version of the manuscript. LP participated in the conception of the study, designed and executed experiments, analyzed and interpreted the data, and wrote the manuscript. SL participated in the conception of the study and in scientific discussions. EH participated in the design and execution of experiments, analysis and interpretation of data, and in scientific discussions. JP participated in the design and execution of experiments and in scientific discussions. CA participated in the conception of the study and in scientific discussions. PM-V participated in the design of experiments and in scientific discussions. PB participated in the conception and direction of the study, design of experiments, analysis, and interpretation of data. CM-M participated in the conception and direction of the study, design and execution of experiments, analysis, and interpretation of data.

### Conflict of Interest

The authors declare that the research was conducted in the absence of any commercial or financial relationships that could be construed as a potential conflict of interest.

## Abbreviations

ASCs: adipose tissue-derived stromal cells
WAT: white adipose tissue
PLIN: perilipin
CAV-1: caveolin-1
PPARγ: peroxisome proliferatoractivated receptor γ
SREBP1C: sterol regulatory element-binding protein-1
TNF-α: tumor necrosis factor-α
IL-10: interleukin-10.
