# Supplementary material for: Leptin Induces Proadipogenic and Proinflammatory Signaling in Adipocytes
Source: Front Endocrinol (Lausanne). 2019 Dec 13;10:841. doi: 10.3389/fendo.2019.00841 (PMC6923660; doi:10.3389/fendo.2019.00841)
Supplement: Supplementary file 1 [file Image_1.pdf]

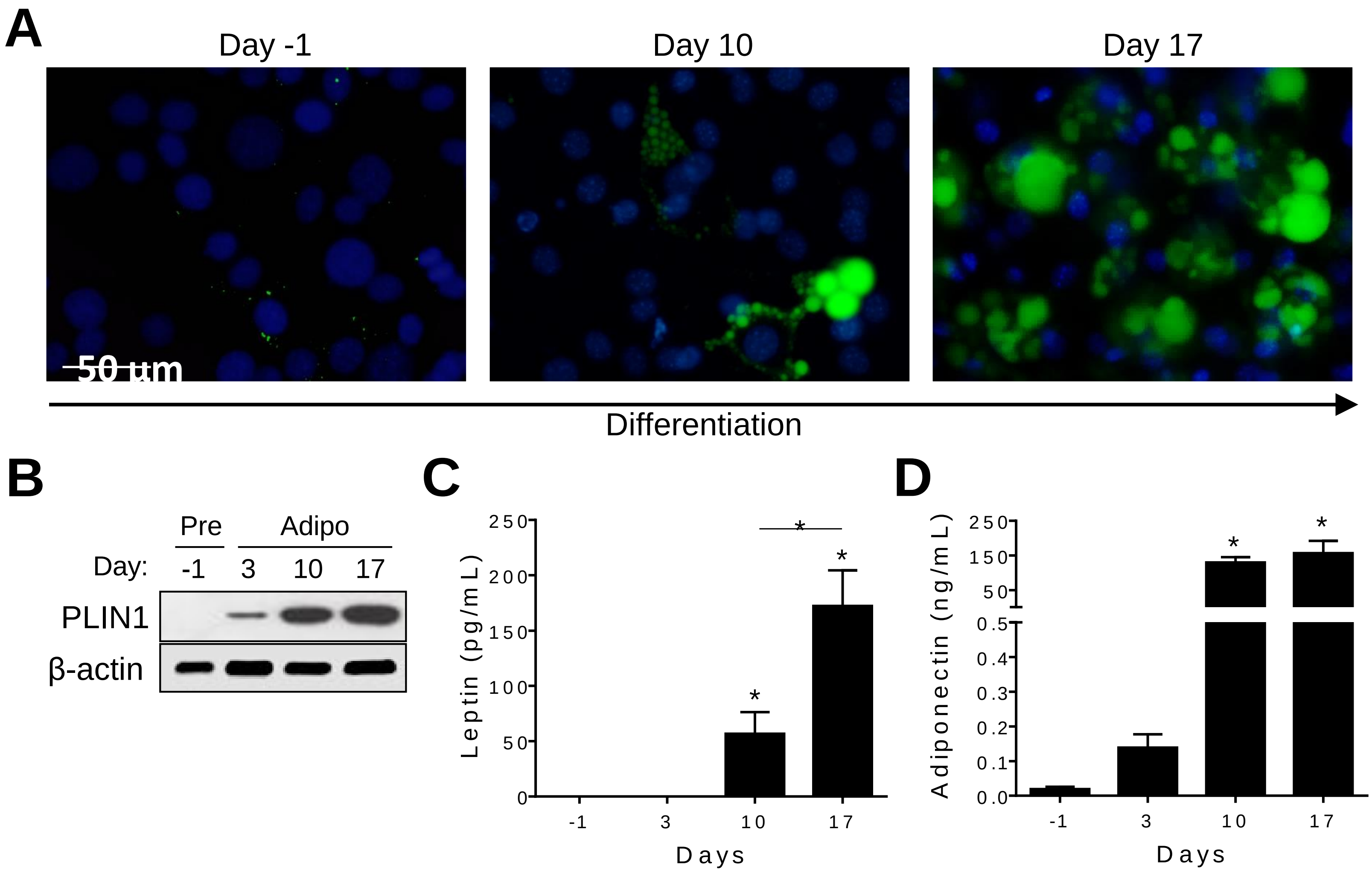

Supplementary Figure 1. 3T3-L1 adipocyte maturation process. (A) Fluorescence microscopic images of 3T3-L1 stained with Bodipy (green) for lipid droplets and DAPI (blue) for nuclei at days -1, 10 and 17. Images are representative of at least 5 independent experiments. **(B)** Western blot analysis of PLIN1, PLIN2 and β-actin in 3T3-L1 preadipocytes (day -1) and adipocytes at 3, 10 and 17 days of differentiation. Blots are representative of 3-5 independent experiments. **(C)** Leptin and **(D)** adiponectin secreted by 3T3-L1 during adipogenesis. Bars represent mean ± standard error of the mean of 7 independent experiments. * = p < 0,01. Statistical analyzes were performed by using Mann-Whitney U tests.
