# Supplementary material for: Leptin Induces Proadipogenic and Proinflammatory Signaling in Adipocytes
Source: Front Endocrinol (Lausanne). 2019 Dec 13;10:841. doi: 10.3389/fendo.2019.00841 (PMC6923660; doi:10.3389/fendo.2019.00841)
Supplement: Supplementary file 2 [file Image_2.pdf]

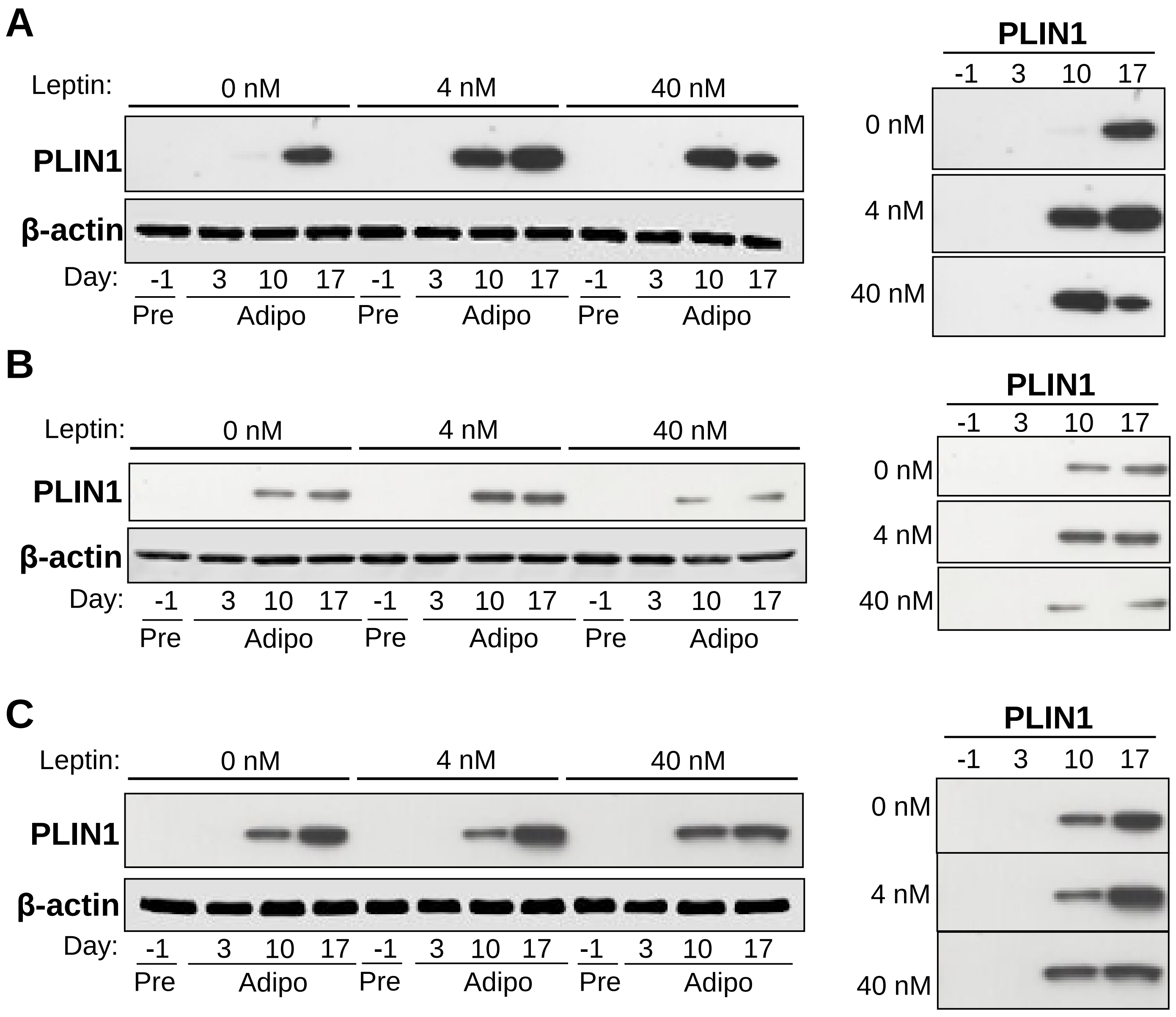

Supplementary Figure 2.

Replicates Figure 1

A-C

PLIN1 replicates relative to figure 1D. 3T3-L1 preadipocytes (day -1) and adipocytes (days 3, 10 and 17) were trated with 0, 4 or 40 nM of leptin during all the culture period and lysates were analysed by western blot. Blots represent 3 independent experiments and are cropped on the right panels for clearer comparison among groups.

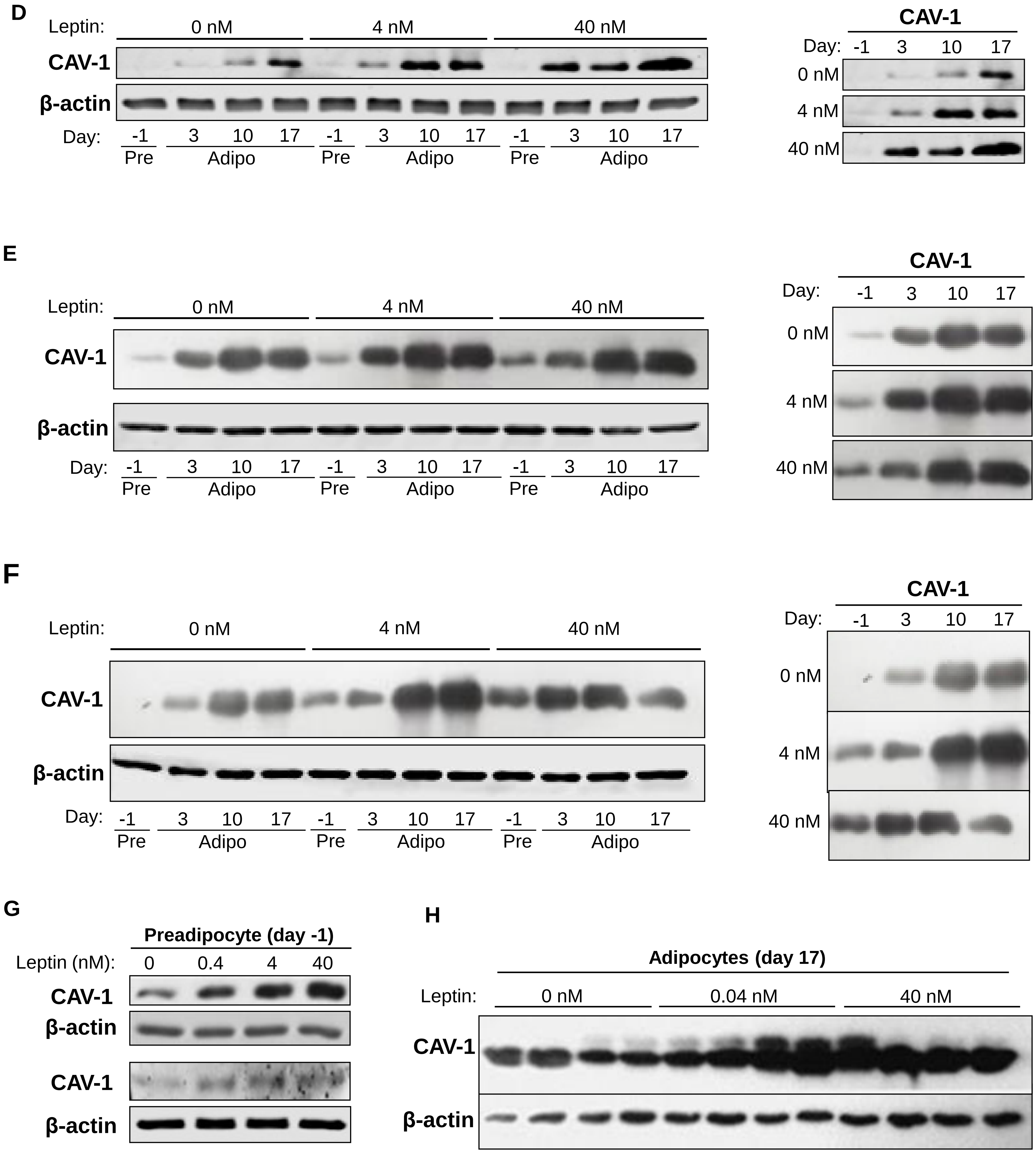

Replicates Figure 1
D-H
CAV-1 replicates relative to figure 1D. 3T3-L1 preadipocytes (day -1) and adipocytes (days 3, 10 and 17) were treated with 0, 0.04, 0.4, 4 or 40 nM of leptin during all the culture period and lysates were analysed by western blot. Blots are from 5 independent experiments and are cropped on the right for clearer comparison among groups. In **G** are shown 2 independent experiments.

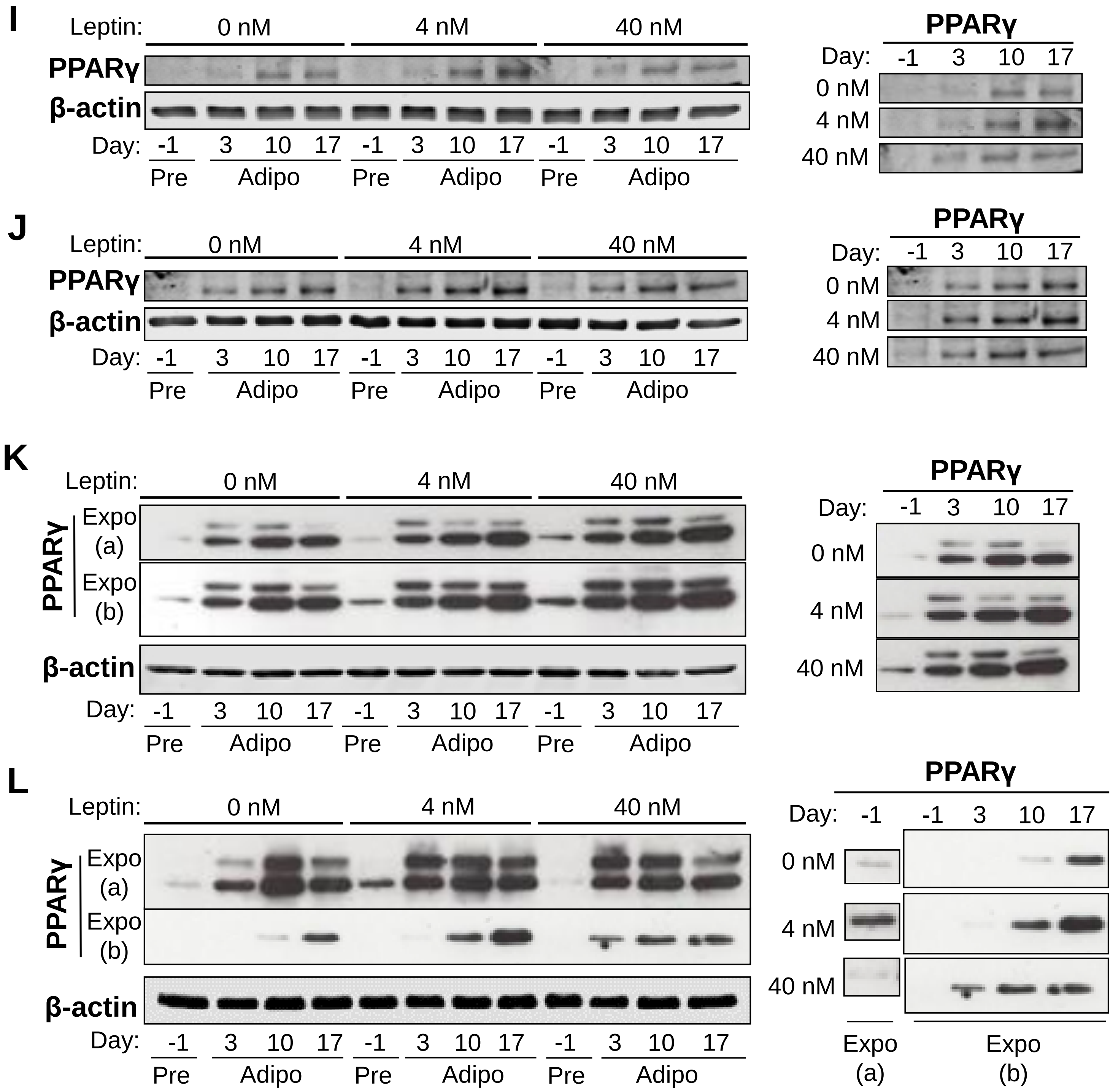

Replicates Figure 1
I-L

. PPARγ replicates relative to figure 1D. 3T3-L1 preadipocytes (day -1) and adipocytes (days 3, 10 and 17) were trated with 0, 4 or 40 nM of leptin during all the culture period and lysates were analysed by western blot. Blots are from 4 independent experiments, and are cropped on the right panels for clearer comparison among groups. "Expo" means exposition time.

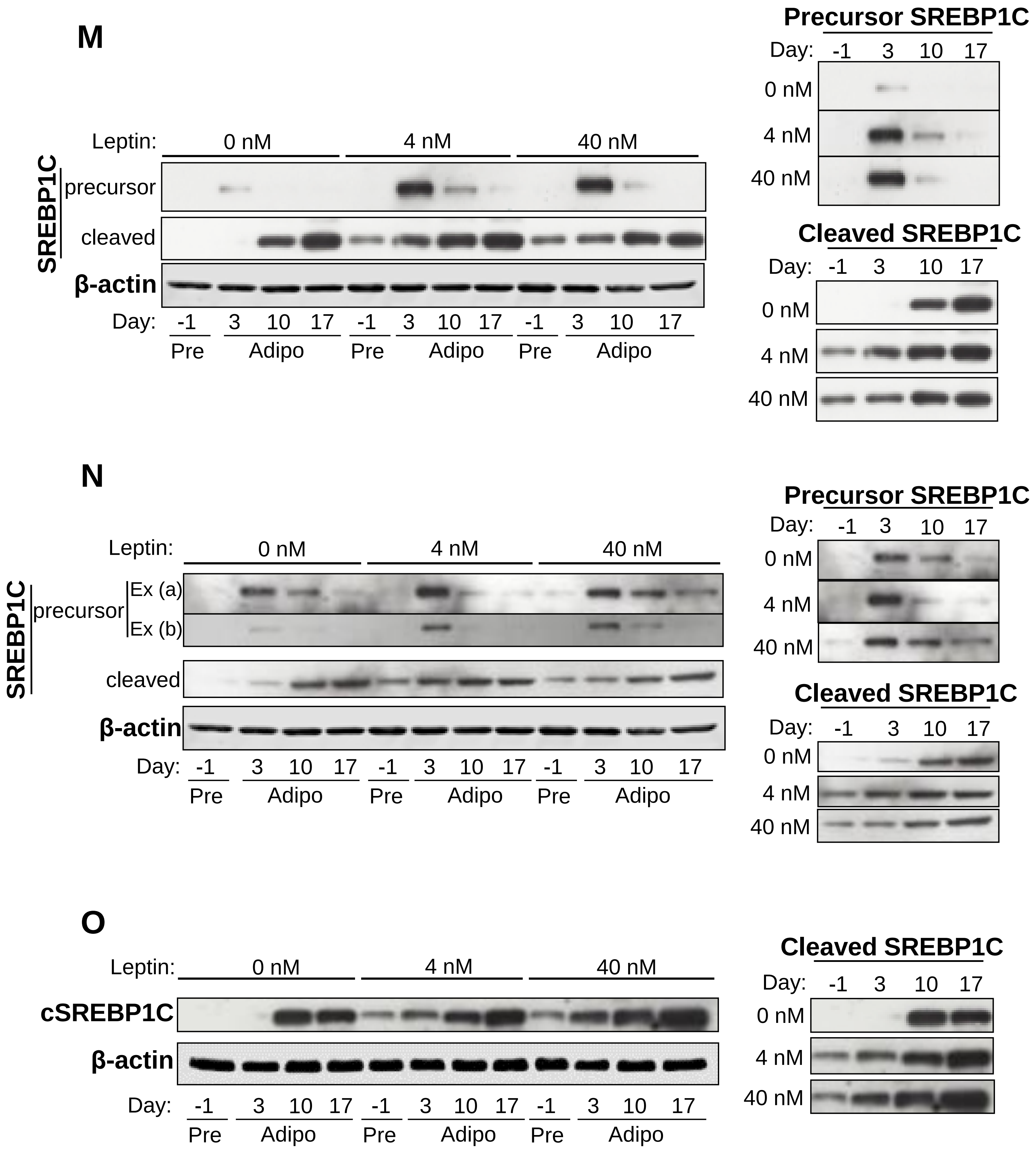

Replicates Figure 1
M-O

SREBP1C replicates relative to figure 1D. 3T3-L1 preadipocytes (day -1) and adipocytes (days 3, 10 and 17) were trated with 0, 4 or 40 nM of leptin during all the culture period and lysates were analysed by western blot. Blots are from 3 independent experiments and are cropped on the right panels for clearer comparison among groups. "Ex" means exposition time.

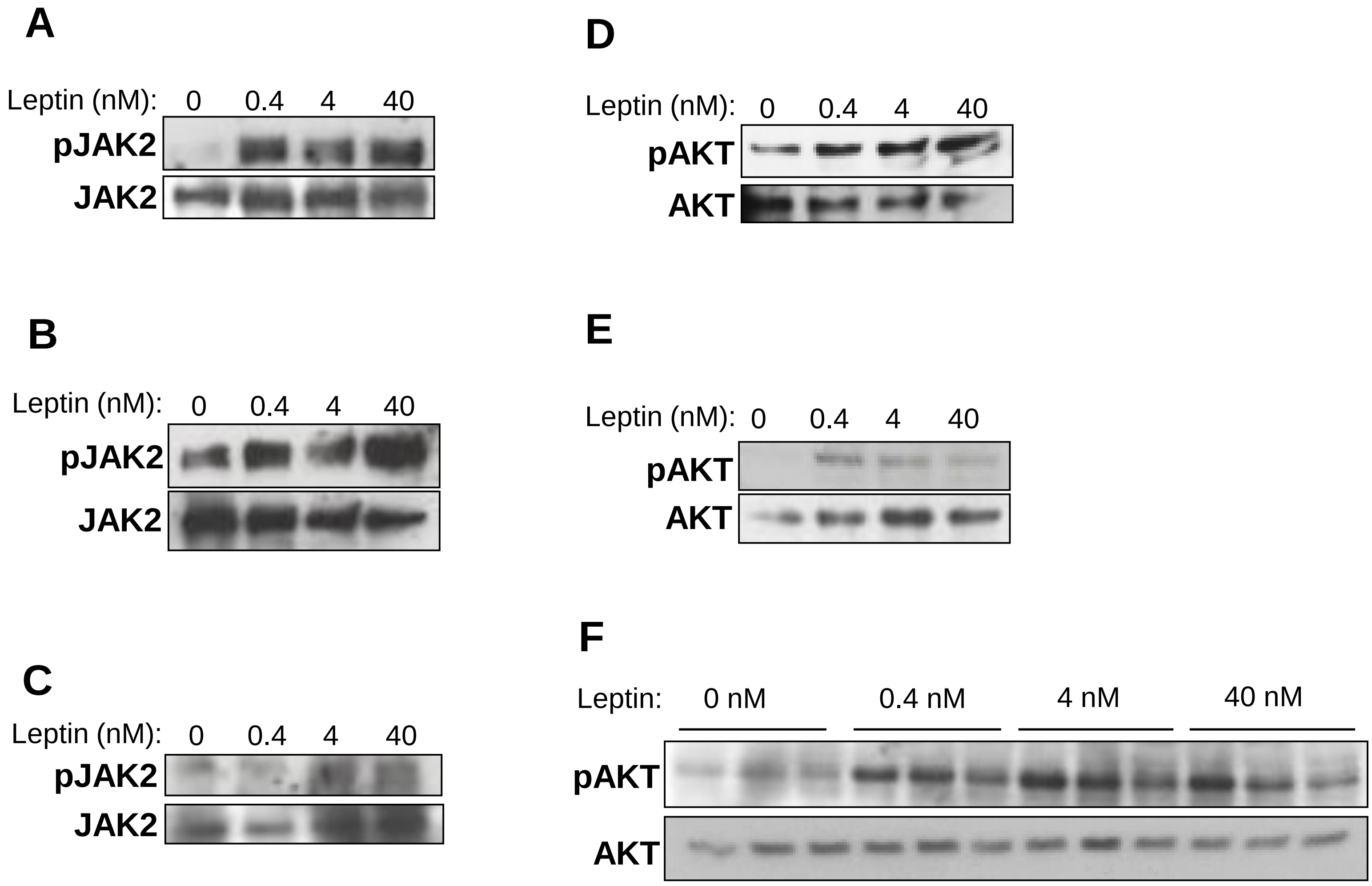

Replicate Figure 2

Phospho JAK2 (pJAK2), total JAK2 (JAK2), phospho AKT (pAKT) and total AKT (AKT) replicates relative to Figure 2A. 3T3-L1 cells were differentiated up to 17 days and then stimulated for 20 min with the indicated concentrations of leptin. Phosphorilation of **(A-C)** JAK2 and **(D-F)** AKT were evaluated by western blot from 3 different experiments (

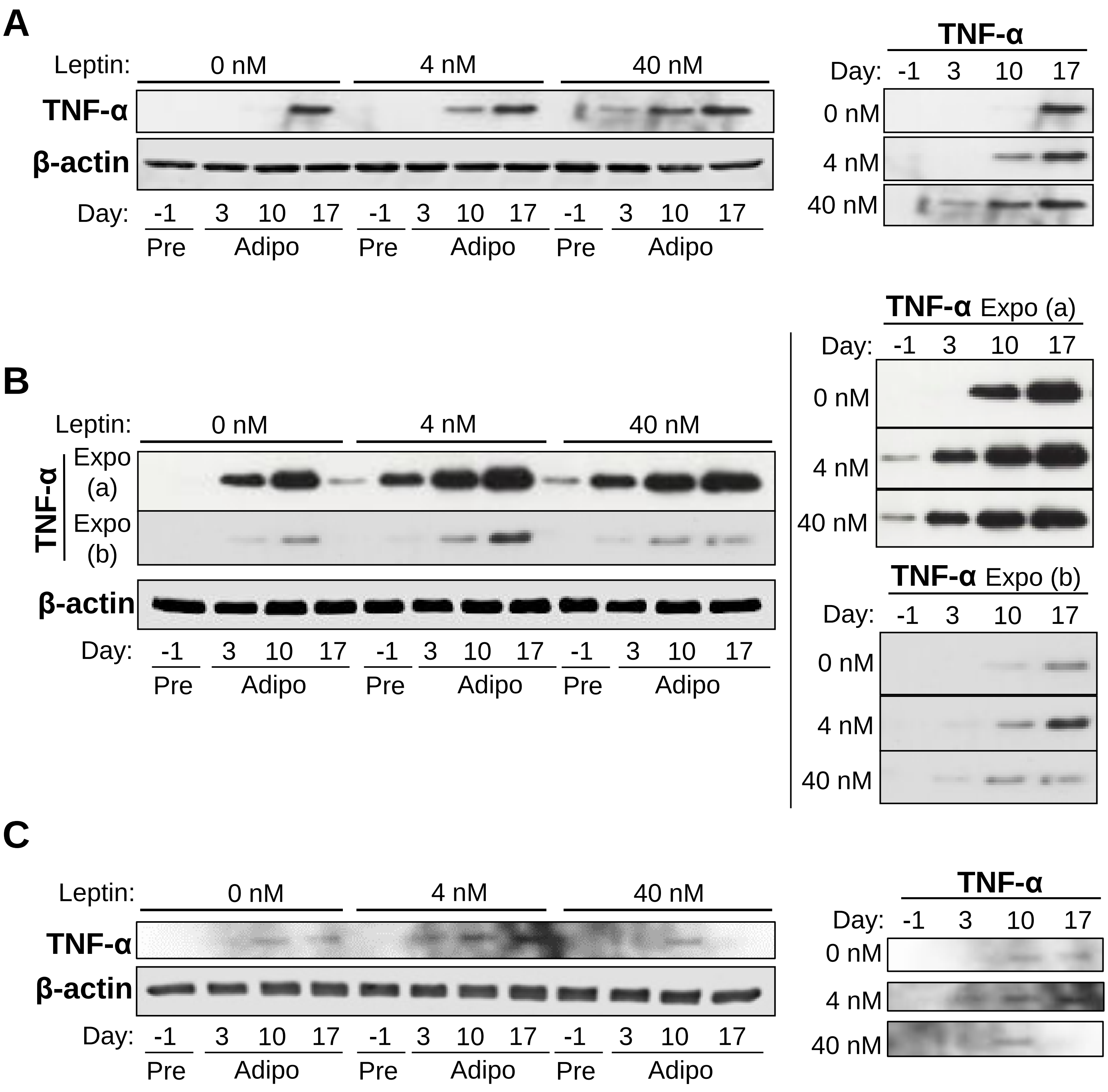

Replicate Figure 3

TNF-α replicates relative to figure 3A. 3T3-L1 preadipocytes (day -1) and adipocytes (days 3, 10 and 17) were trated with 0, 4 or 40 nM of leptin during all the culture period and lysates were analysed by western blot. Blots are from 3 independent experiments and are cropped on the right panels for clearer comparison among groups. "Expo" means exposition time.

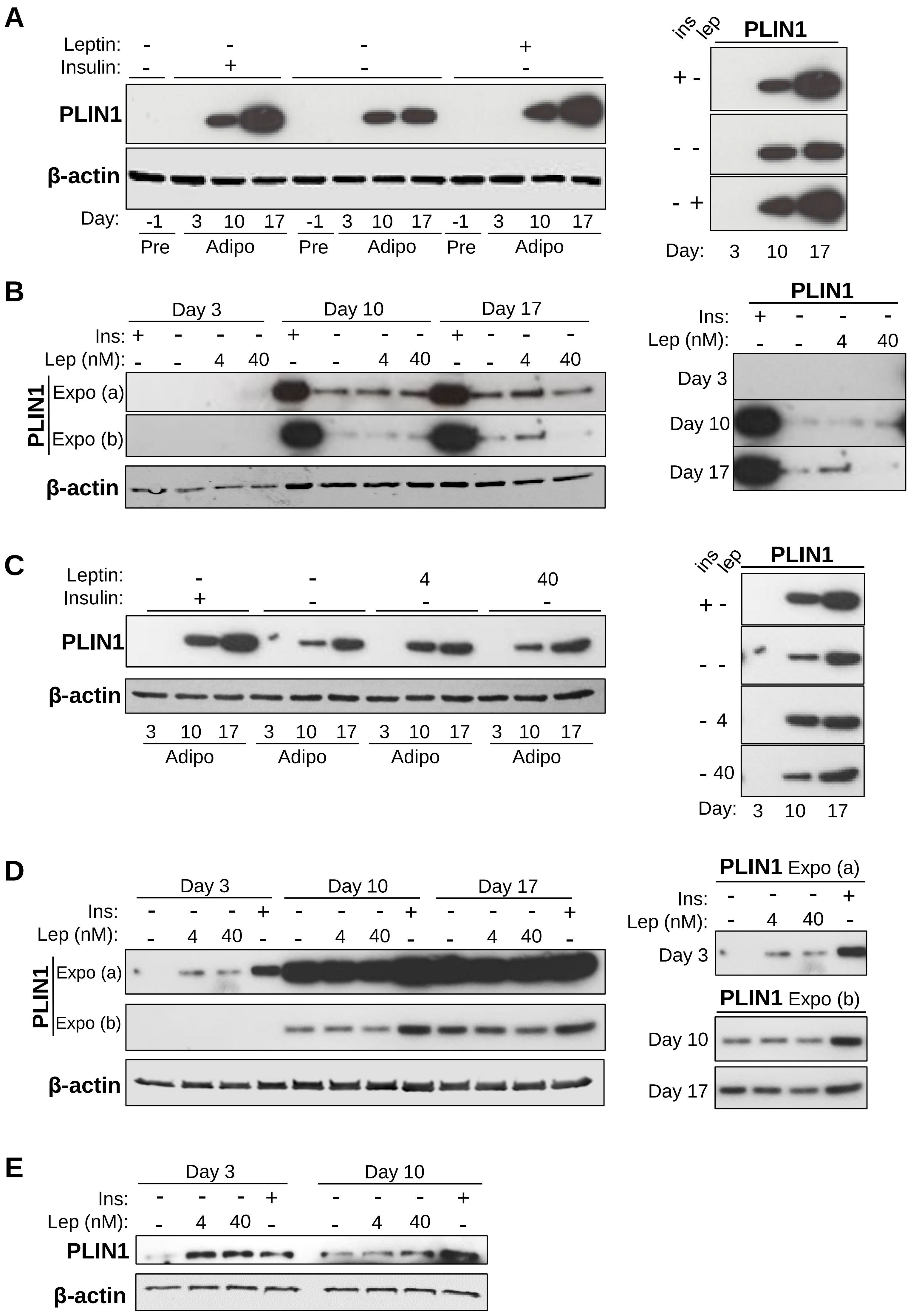

Replicates Figure 4

A-E

PLIN1 replicates relative to figure 4C. (A-E) 3T3-L1 preadipocytes (day -1) and adipocytes (days 3, 10 and 17) were trated with 0, 4 or 40 nM of leptin, in the presence or absence of insulin from day 0 on. Lysates were analysed by western blot. Blots are from 5 independent experiments and are cropped on the right for clearer comparison among groups. "Expo" means exposition time.

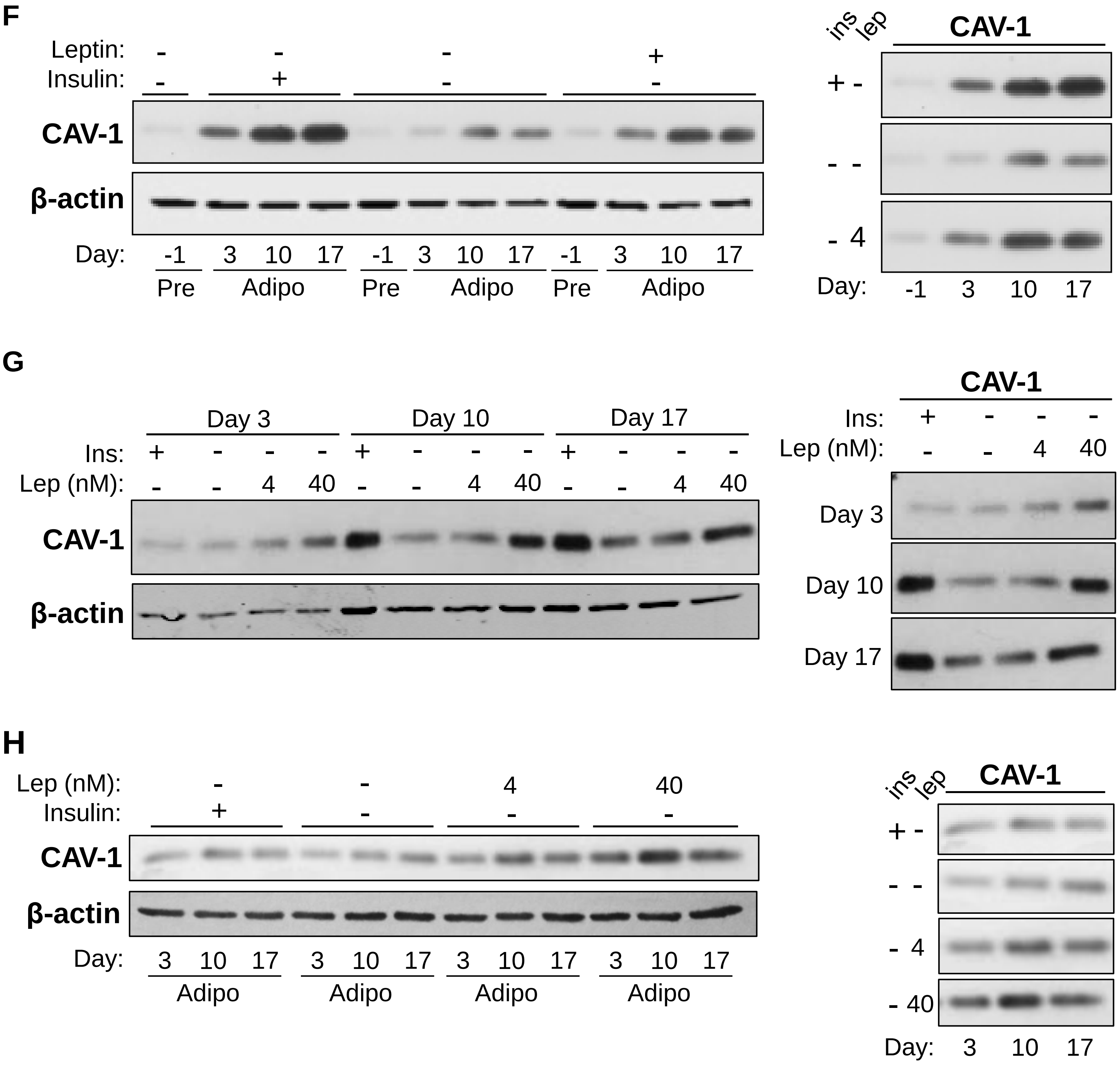

Replicates Figure 4

F-H

CAV-1 replicates relative to figure 4C. 3T3-L1 preadipocytes (day -1) and adipocytes (days 3, 10 and 17) were trated with 0, 4 or 40 nM of leptin, in the presence or absence of insulin from day 0 on. Lysates were analysed by western blot. Blots are from 3 independent experiments and are cropped on the right for clearer comparison among groups.

I

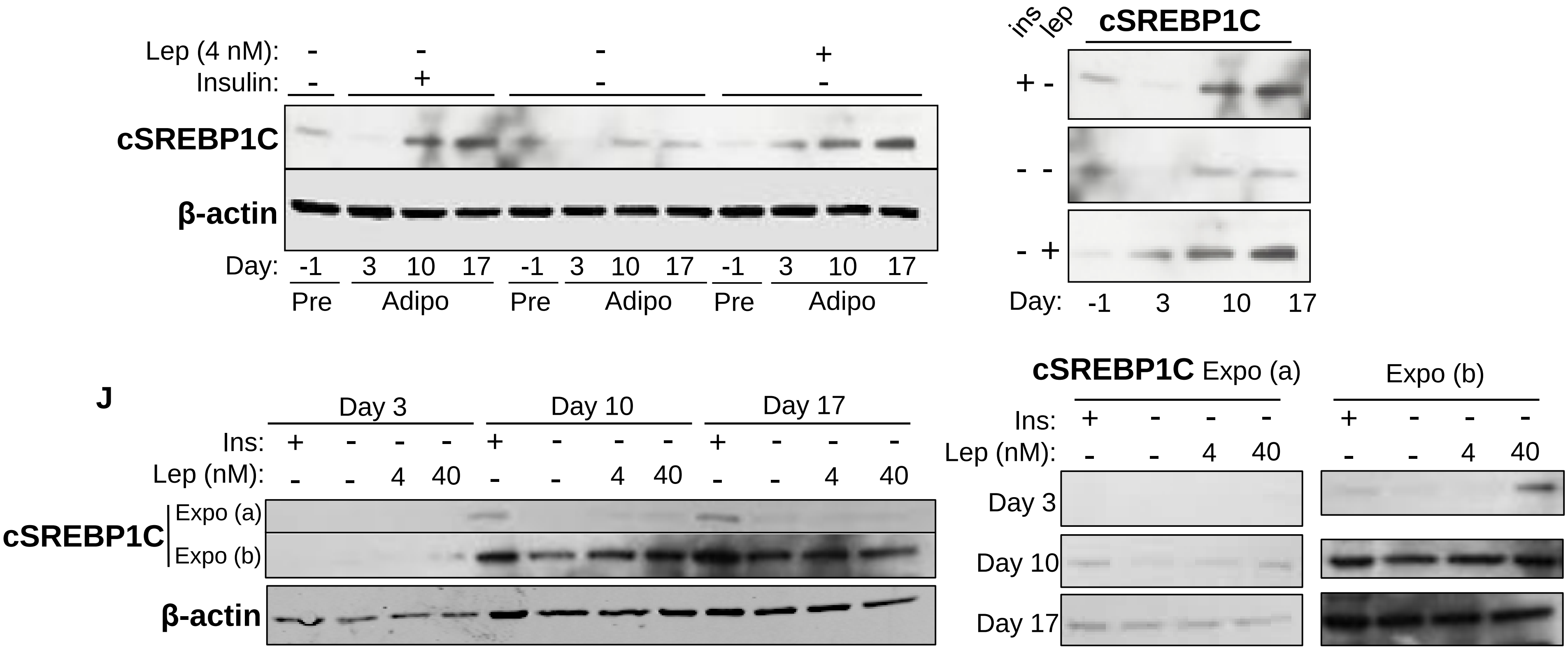

Replicates Figure 4
I-J
cSREBP1C replicates relative to figure 4C. (A-C) 3T3-L1 preadipocytes (day -1) and adipocytes (days 3, 10 and 17) were trated with 0, 4 or 40 nM of leptin, in the presence or absence of insulin from day 0 on. Lysates were analysed by western blot. Blots are from 2 independent experiments and are cropped on the right for clearer comparison among groups.

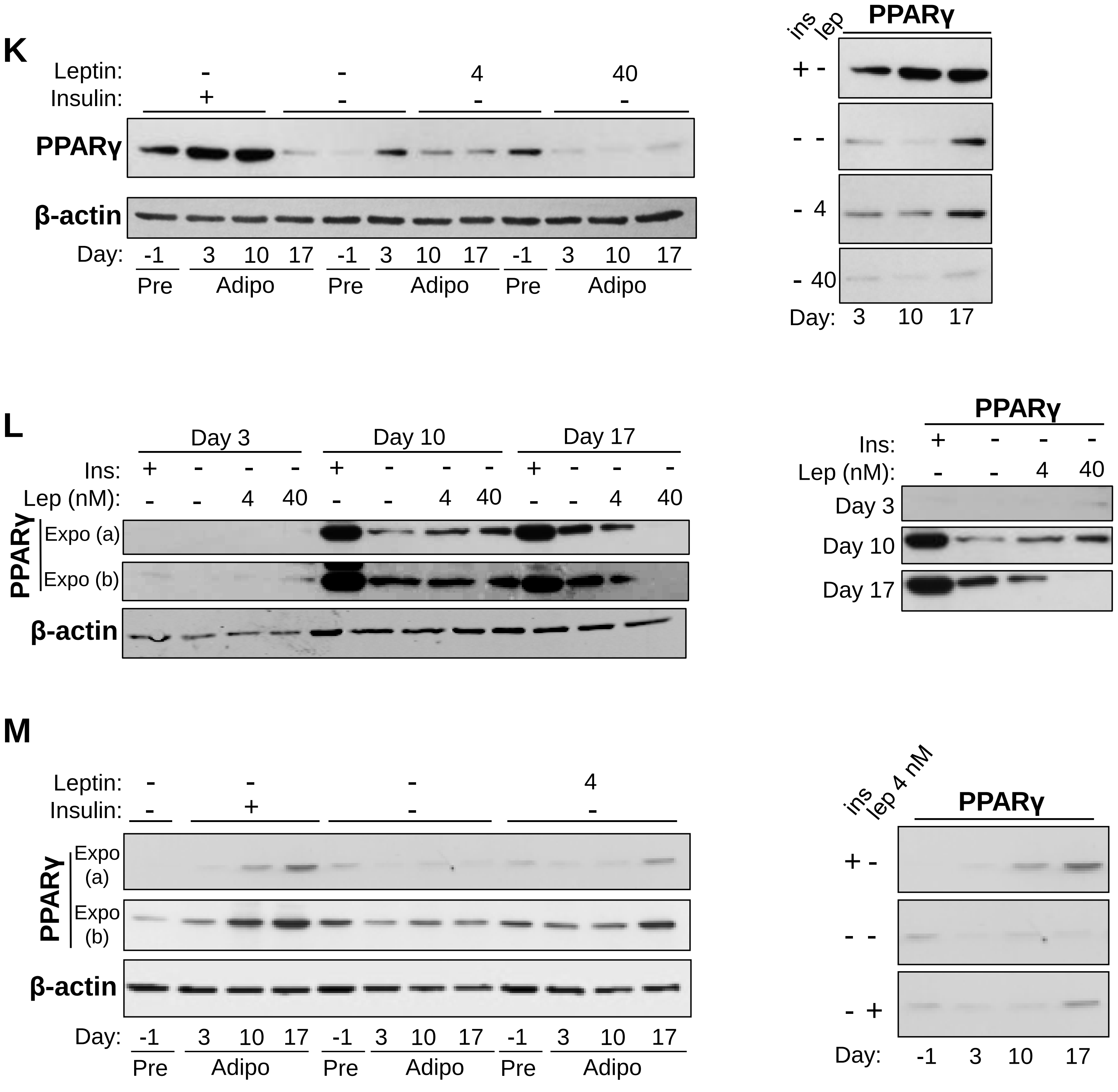

Replicates Figure 4

K-M

PPARγ replicates relative to figure 4C. (A-C) 3T3-L1 preadipocytes (day -1) and adipocytes (days 3, 10 and 17) were trated with 0, 4 or 40 nM of leptin, in the presence or absence of insulin from day 0 on. Lysates were analysed by western blot. Blots are from 2 independent experiments and are cropped on the right for clearer comparison among groups.

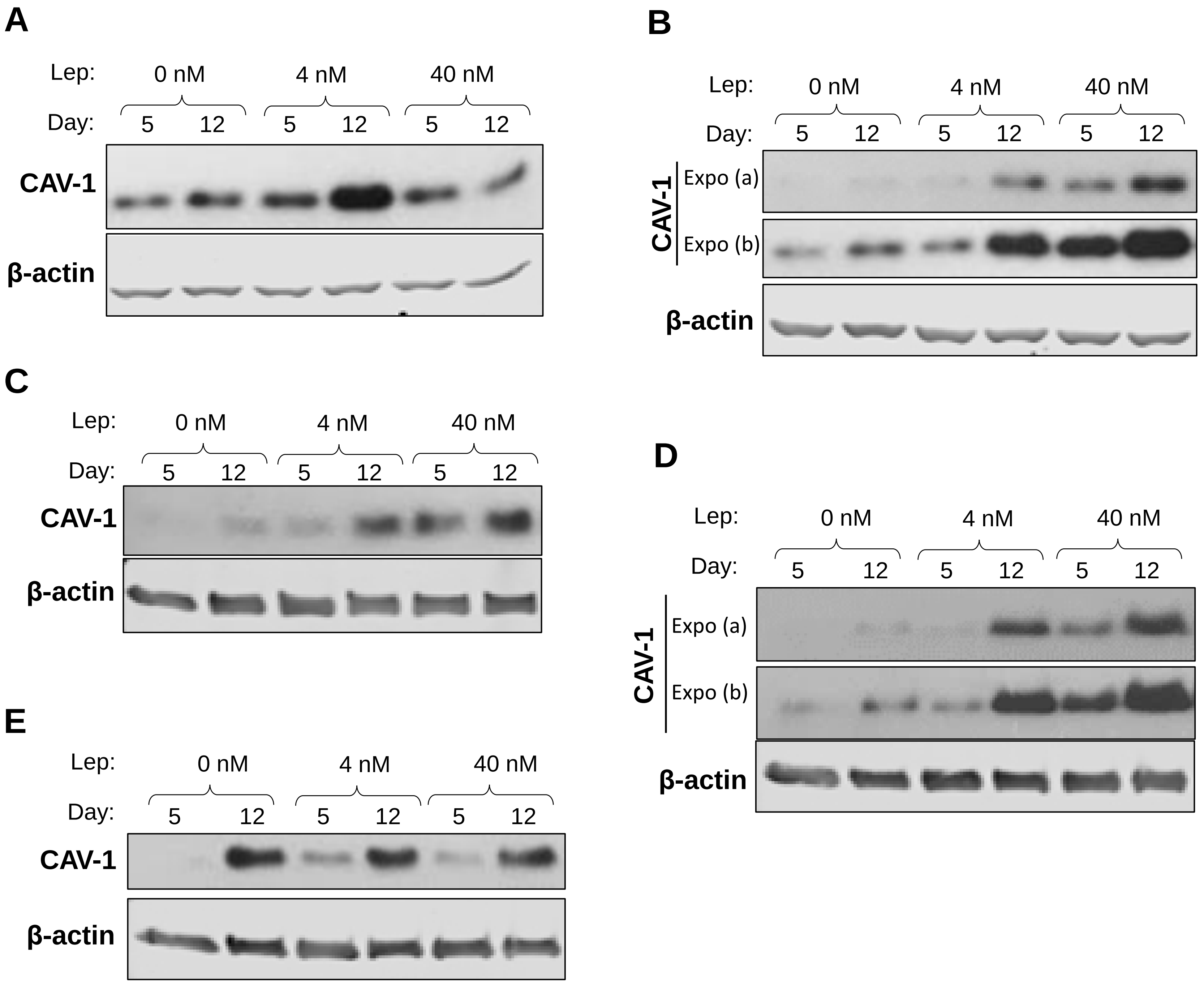

Replicates Figure 5
A-E.

CAV-1 replicates relative to figure 5C. Retroperitoneal ASCs differentiated into adipocytes for 5 or 12 days were trated with 0, 4 or 40 nM of leptin during all the culture period. Lysates were analysed by Western blot for CAV-1. Blots are from 5 independent experiments as can be followed by the β-actin lanes. "Expo" means exposition time.

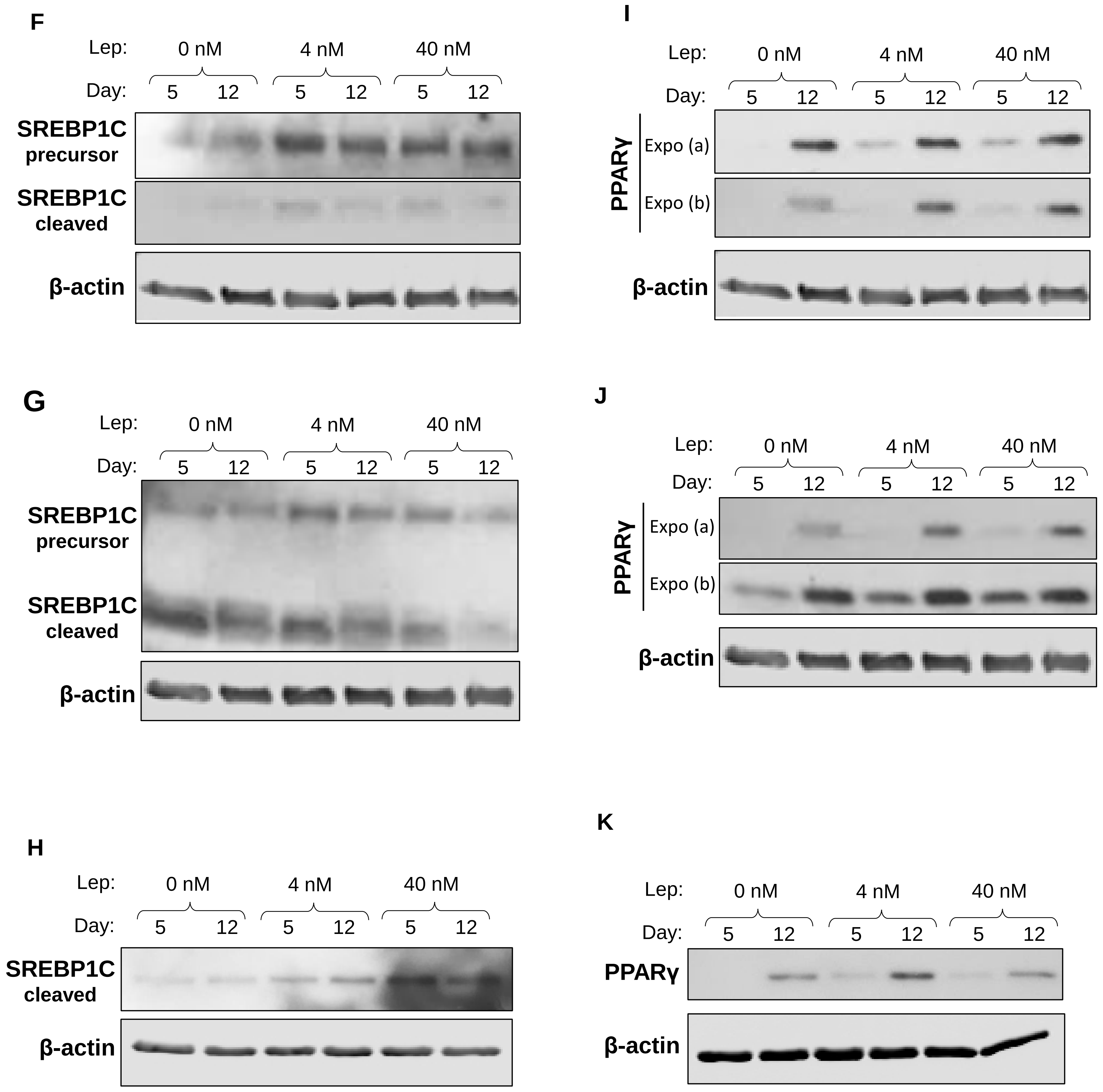

Replicates Figure 5
F-K.

SREBP1C and PPARγ replicates relative to figure 5C. Retroperitoneal ASCs differentiated into adipocytes for 5 or 12 days were trated with 0, 4 or 40 nM of leptin during all the culture period. Lysates were analysed by Western blot for SREBP1C **(F-H)** and for PPARγ **(I-K)** . Blots are from 4 independent experiments and some blots were done from the same samples/membranes as can be seen by the β-actin similar lanes (2 pairs A /D; B/E, C and F are from 2 other experiments). "Expo" means exposition time.

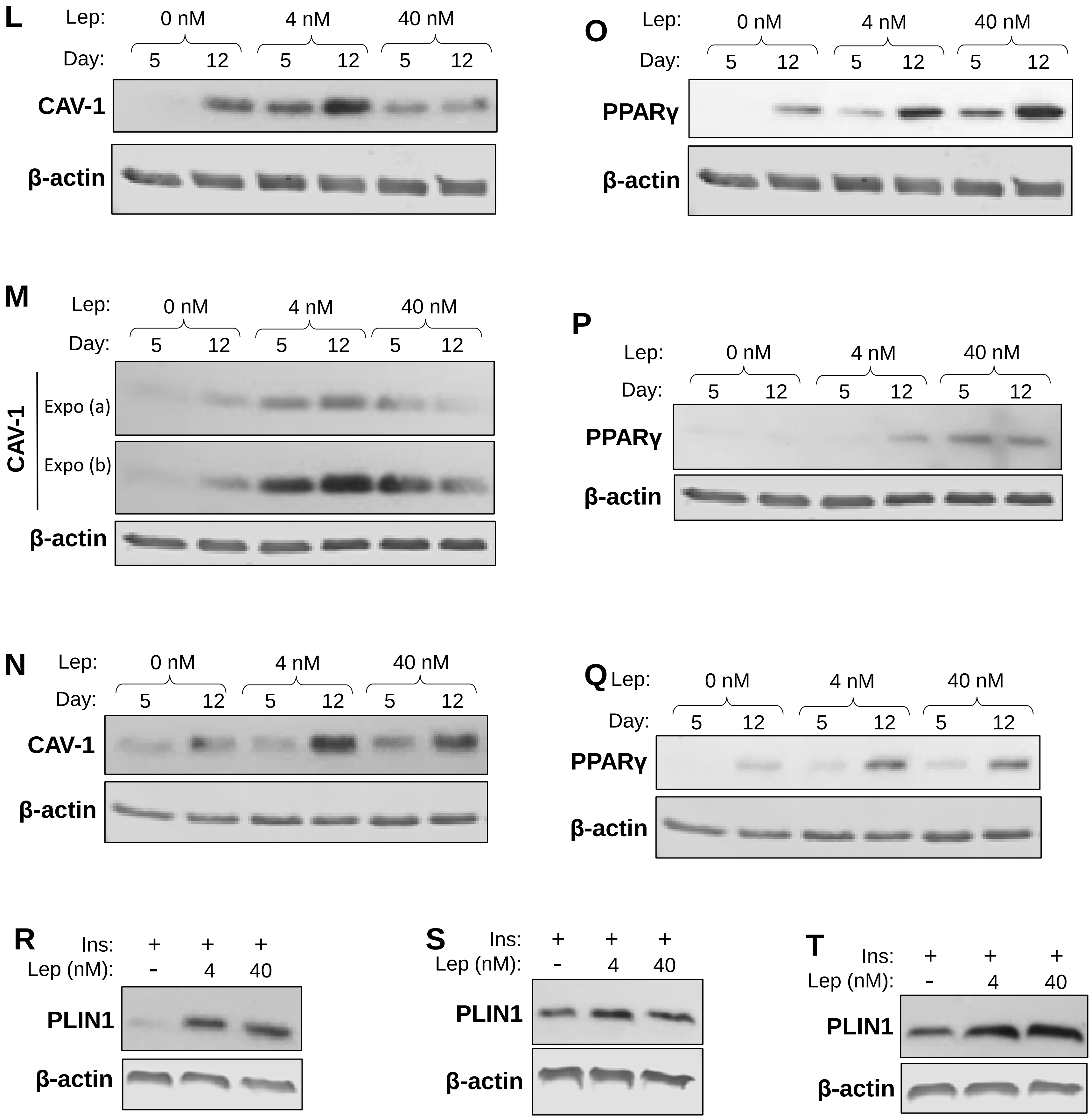

Replicates Figure 5

L-T.

CAV-1, PPARγ and PLIN1 replicates relative to figure 5D. Subcutaneous ASCs differentiated into adipocytes for 5 or 12 days were trated with 0, 4 or 40 nM of leptin during all the culture period **(L-Q)**. Adipocytes with 12 days of differentiation **(R-T)** . Lysates were analysed by Western blot for the expression of CAV-1 **(L-N)** , PPARγ **(O-Q)** and PLIN1 **(R-T)** . Blots are from 3 independent experiments.

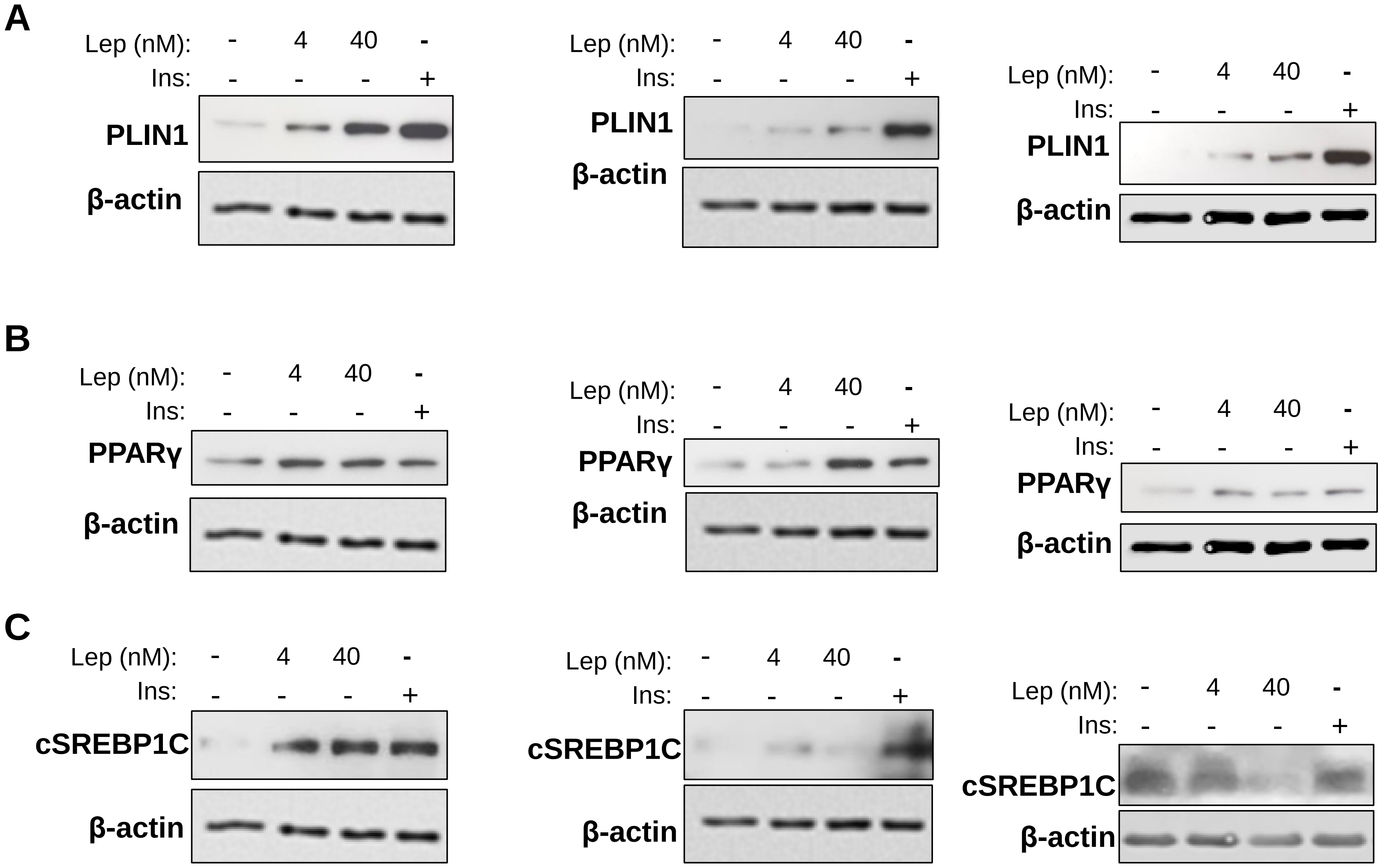

Replicates Figure A-C.

PPARγ, cSREBP1C and PLIN1 replicates relative to figure 7D. Subcutaneous ASCs were differentiated into adipocytes for 5 days and trated with 0, 4 or 40 nM of leptin, in the presence or absence of insulin from day 0 on. Lysates were analysed by Western blot for the expression of PLIN1 **(A)**, PPARγ **(B)** and cSREBP1C **(C)**. Blots represent 3 independent experiments and same the last panel (cSREBP1C) is from a different experiment.

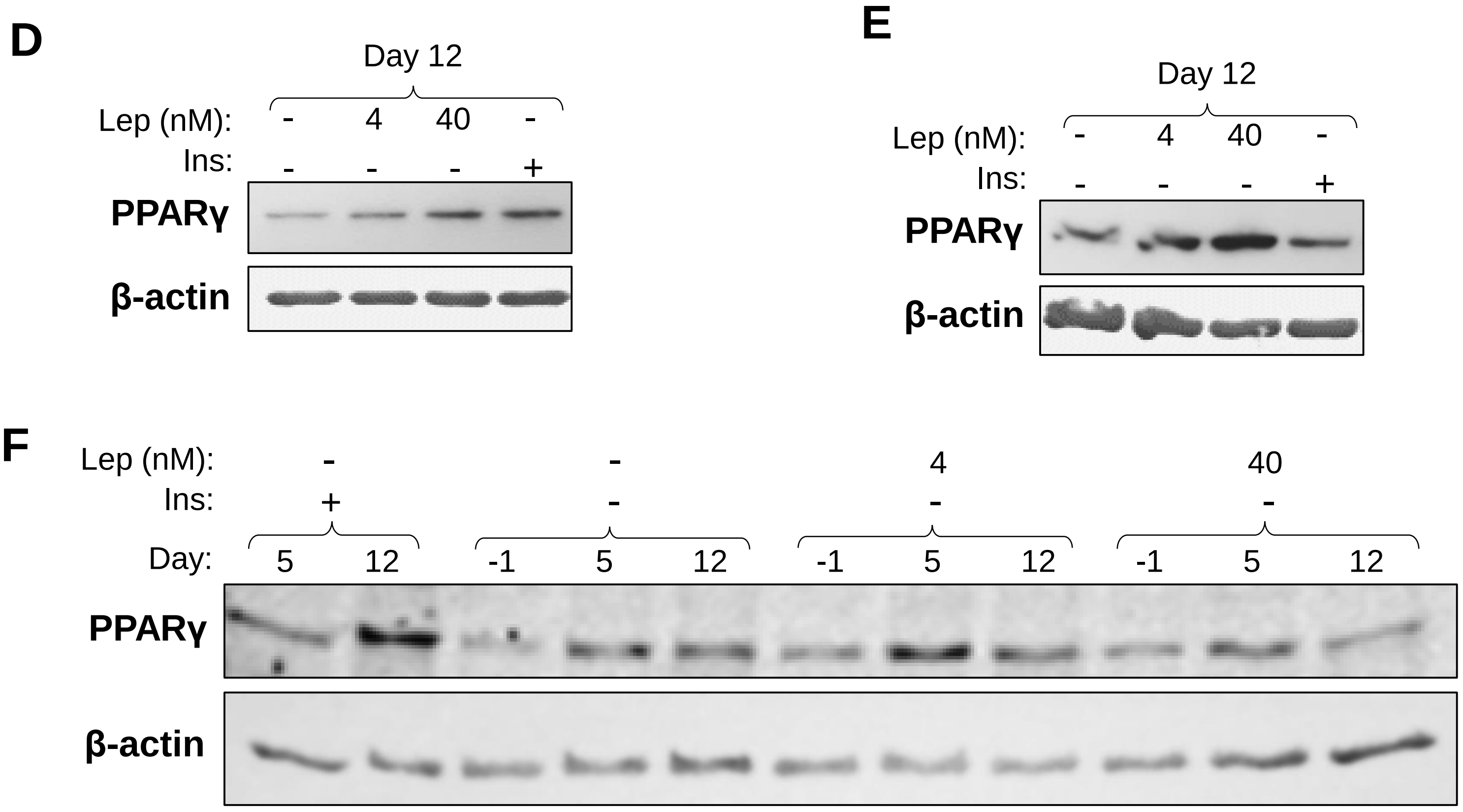

Replicates Figure 7
D-F.

PPARγ replicates relative to figure 7E. Retroperitoneal ASCs differentiated into adipocytes for 5 or 12 days were trated with 0, 4 or 40 nM of leptin, in the presence or absence of insulin from day 0 on. Lysates were analysed by Western blot for PPARγ expression. Blots are from 3 independent experiments.

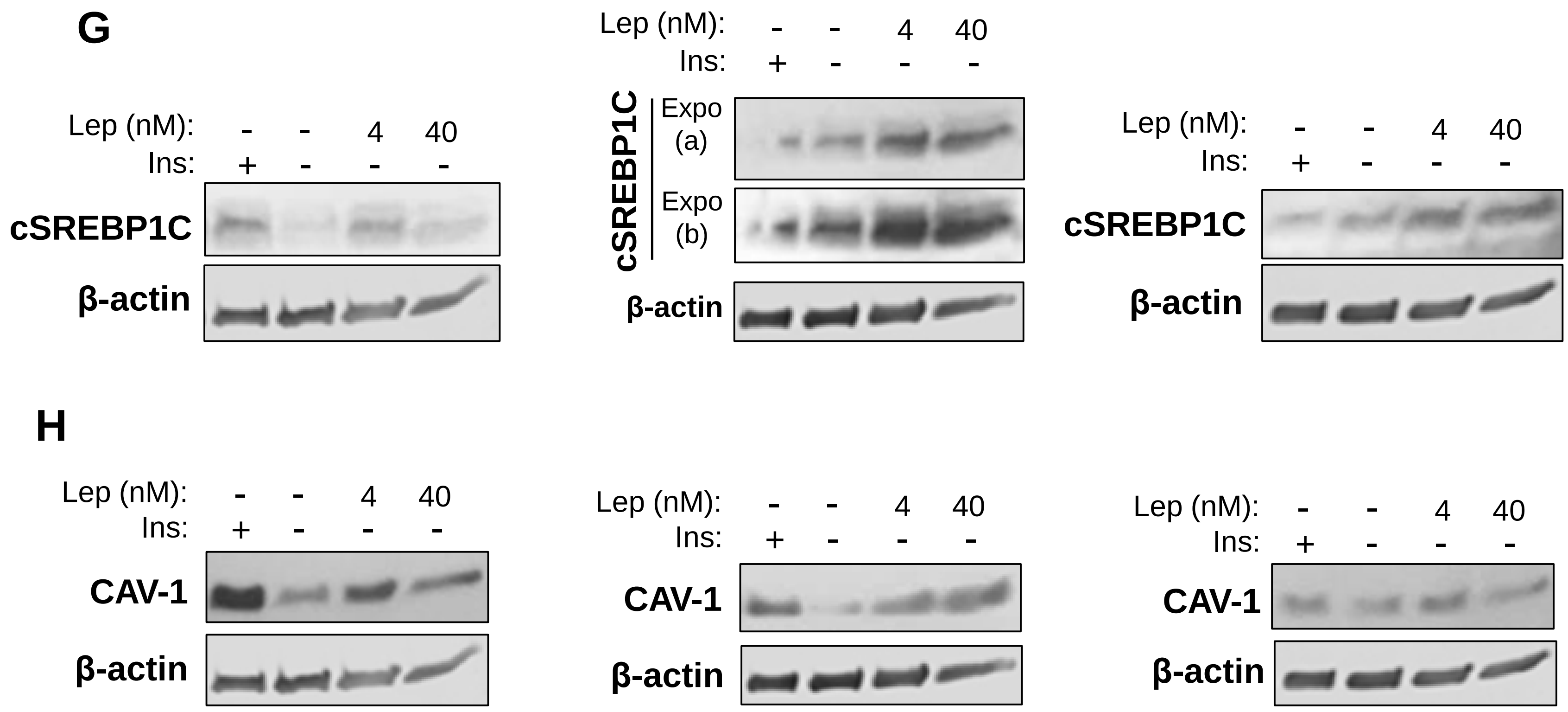

Replicates Figure 7
G-H.
cSREBP1C and CAV-1 replicates relative to figure 7E. Retroperitoneal ASCs differentiated into adipocytes for 12 days were trated with 0, 4 or 40 nM of leptin, in the presence or absence of insulin from day 0 on. Lysates were analysed by Western blot for the expression of CAV-1 **(G)** and cSREBP1C **(H)**. Blots are from 3 independent experiments.
