# Supplementary figures and images for: Leptin Induces Proadipogenic and Proinflammatory Signaling in Adipocytes
Source: Front Endocrinol (Lausanne). 2019 Dec 13;10:841. doi: 10.3389/fendo.2019.00841 (PMC6923660; doi:10.3389/fendo.2019.00841)

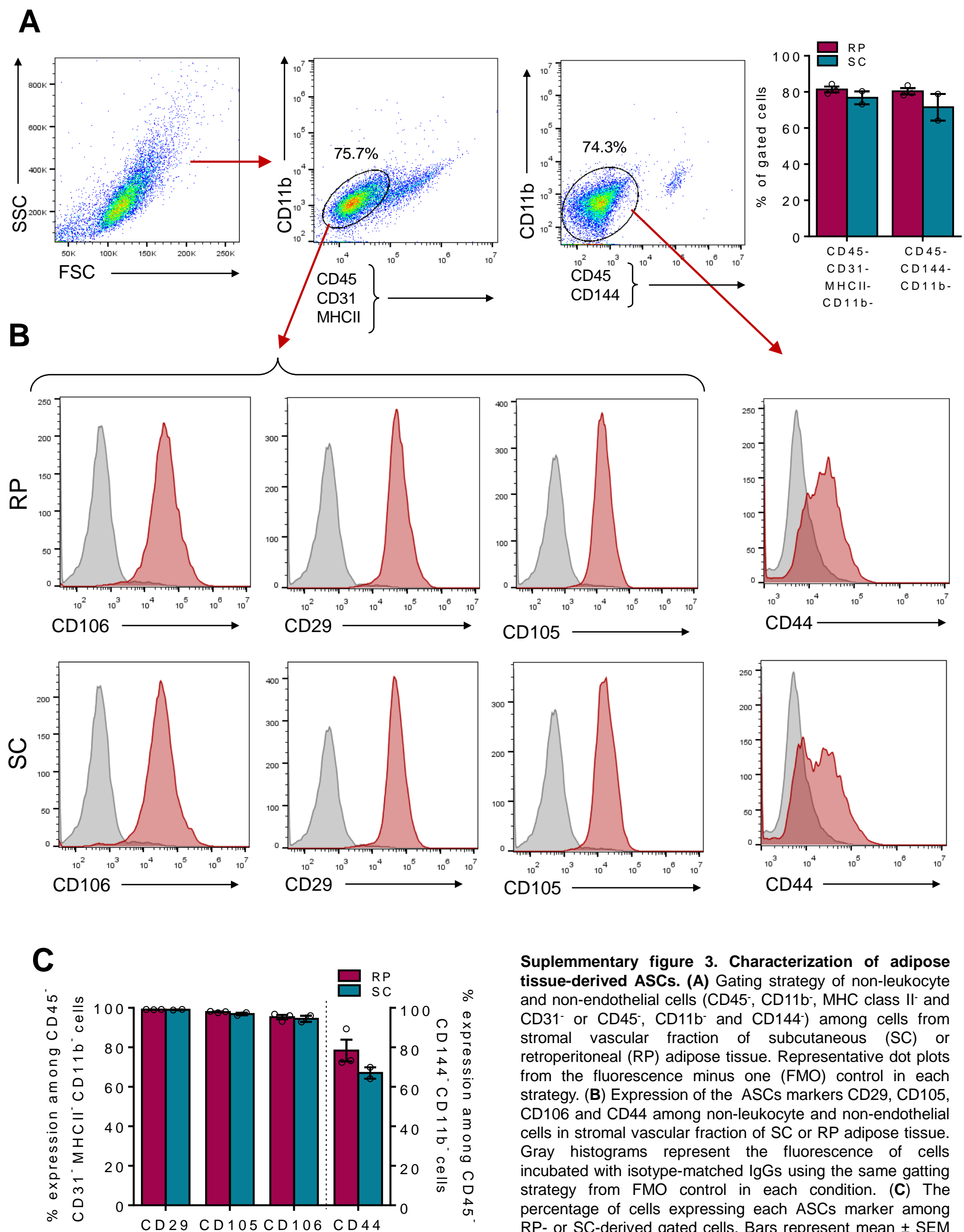

Supplement: Supplementary file 3 [file Image_3.pdf]
