# Supplementary material for: Leptin Induces Proadipogenic and Proinflammatory Signaling in Adipocytes
Source: Front Endocrinol (Lausanne). 2019 Dec 13;10:841. doi: 10.3389/fendo.2019.00841 (PMC6923660; doi:10.3389/fendo.2019.00841)
Supplement: Supplementary file 4 [file Image_4.pdf]

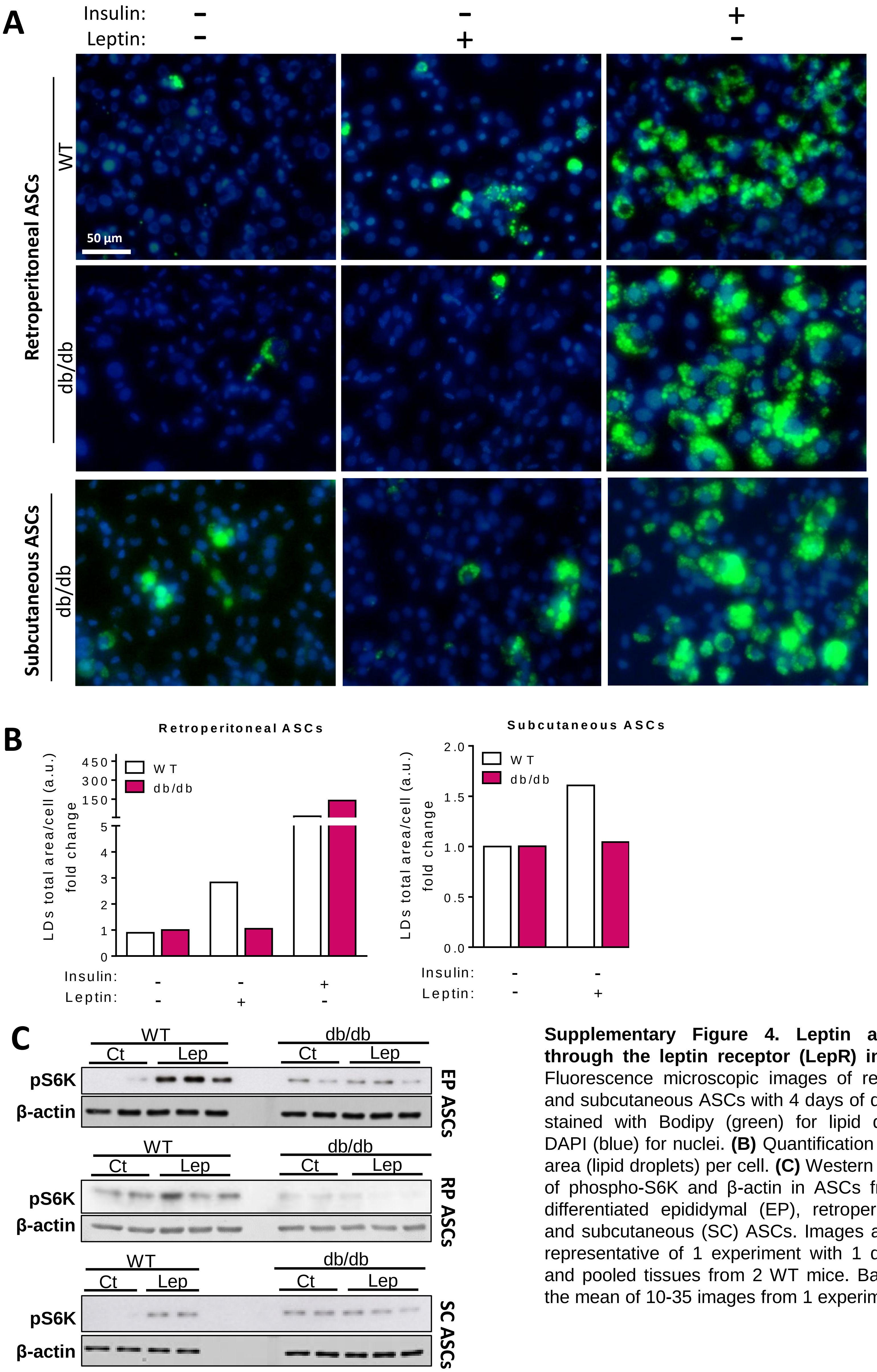

Supplementary Figure 4. Leptin acts strictly through the leptin receptor (LepR) in ASCs. (A) Fluorescence microscopic images of retroperitoneal and subcutaneous ASCs with 4 days of differentiation stained with Bodipy (green) for lipid droplets and DAPI (blue) for nuclei. **(B)** Quantification of the green area (lipid droplets) per cell. **(C)** Western blot analysis of phospho-S6K and β-actin in ASCs from 4 days-differentiated epididymal (EP), retroperitoneal (RP) and subcutaneous (SC) ASCs. Images and blots are representative of 1 experiment with 1 db/db mouse and pooled tissues from 2 WT mice. Bars represent the mean of 10-35 images from 1 experiment.
